# Porphyrin-decorated perylene–nickel-MOF nanocomposite: photoinduced electron and energy transfer for photoelectrochemical applications

**DOI:** 10.1039/d6ra09222e

**Published:** 2026-11-09

**Authors:** Hadeer M. Mostafa, Ayman A. Abdel-Shafi, Sherif F. Hammad, Haitham M. El-Bery, Mohamed E. El-Khouly

**Affiliations:** a Nanoscience Program, Faculty of Basic and Applied Sciences, Egypt-Japan University of Science and Technology (E-JUST) New Borg El-Arab City Alexandria Egypt; b Department of Chemistry, Faculty of Science, Qena University Qena Egypt; c Department of Chemistry, Faculty of Science, Ain Shams University 11566 Abbassia Cairo Egypt; d Pharm D Program, Egypt-Japan University of Science and Technology New Borg El-Arab City Alexandria Egypt; e Chemistry Department, Faculty of Science, Assiut University Assiut 71515 Egypt; f Science & Innovation Center of Excellence, Egyptian Russian University Badr City 11829 Cairo Egypt mohamed.elkhouly@ejust.edu.eg

## Abstract

Developing aqueous supramolecular light-harvesting systems that combine broad solar spectrum absorption with efficient photoinduced electron and energy transfer remains a significant challenge for sustainable energy production. To address this goal, a novel supramolecular light-harvesting system was constructed in an aqueous medium *via* electrostatic interactions between porphyrin-5,10,15,20-tetrakis(1-methylpyridin-1-ium) 4-methylbenzenesulfonate (TMPyP) and perylene-based nickel MOF nanosheets (TMPyP/Ni-PTCA-SDS). The structure and morphology of the system were investigated using various spectroscopic techniques. Steady-state absorption measurements demonstrated that the TMPyP/Ni-PTCA-SDS nanosheets possess high light-harvesting efficiency across a broad range of the solar spectrum, indicating their suitability for light energy conversion. Upon photoexcitation of the TMPyP entity, steady-state and time-resolved fluorescence measurements revealed intra-supramolecular electron transfer from the electron-donating TMPyP to the electron-accepting Ni-PTCA-SDS nanosheets, proceeding with an efficiency of 98% and a rapid rate constant of 1.25 × 10^10^ s^−1^. Conversely, photoexcitation of the Ni-PTCA-SDS component drove Förster resonance energy transfer (FRET) from the singlet state of Ni-PTCA-SDS to TMPyP with an efficiency of 69%. Furthermore, complementary cyclic voltammetry, electrochemical impedance spectroscopy (EIS), and chronoamperometry confirm enhanced interfacial electron transfer and reduced charge-transfer resistance under illumination. These findings provide an effective strategy for constructing stable, aqueous supramolecular platforms featuring efficient photoinduced electron- and energy-transfer relays for light-harvesting and solar energy conversion applications.

## Abstract 2

Developing aqueous supramolecular light-harvesting systems that combine broad solar spectrum absorption with efficient photoinduced electron and energy transfer remains a significant challenge for sustainable energy production.

## Graphical abstract

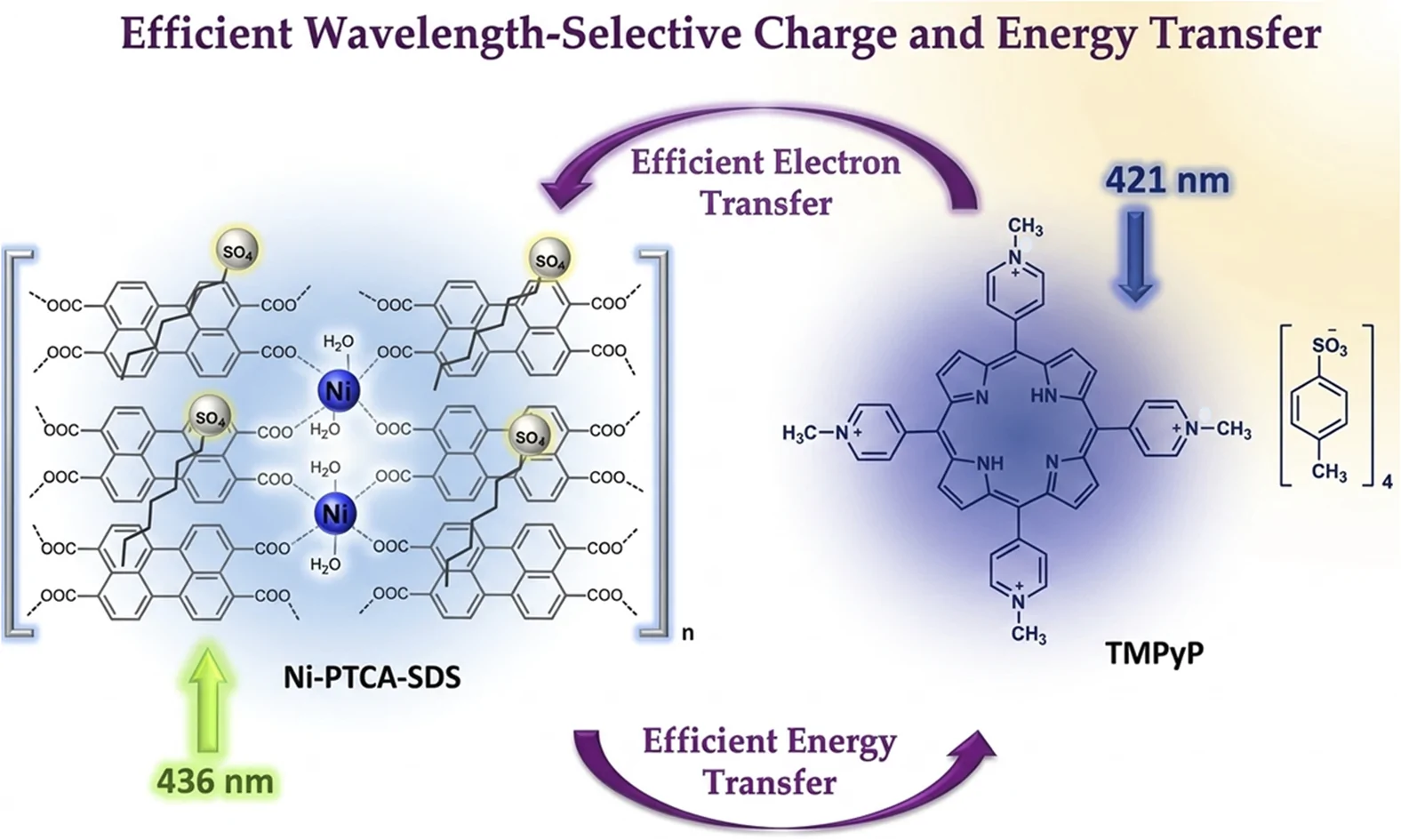

## 1. Introduction

Artificial light-harvesting materials have acquired significant interest as renewable sources inspired by the capability of supramolecular assemblies in natural photosynthesis to capture and convert light energy into chemical energy, paving the way for new technologies and applications.^1–13^ Particularly, the construction of self-assembling nanoarchitectonics emerged as a promising design for the development of light-harvesting systems with remarkable optical properties.^14–20^

Recently, metal–organic frameworks (MOFs), a new class of crystalline molecular materials designed from the interaction of organic ligands with metal or metal clusters, have been applied as a versatile platform for developing light-harvesting systems.^21–28^ The ability to incorporate highly photoactive molecular functional components in micro- or *meso*-porous extended networks nominates MOFs to be functionalized in many potential applications.^29–41^ However, most MOFs have a wide band gap between the highest occupied molecular orbital (HOMO) and the lowest unoccupied molecular orbital (LUMO) which restricts their optical absorption primarily to the ultraviolet (UV) region.^42^ While many molecular and supramolecular light harvesting systems consisting of donor–acceptor (D–A) units have been generally exploited as promising artificial light harvesting components,^1,12,14,15^ systematic developments to boost the working principle and improve the fundamental energy transfer (EnT) and/or photoinduced charge transfer (PCT) processes in MOF-derived systems are rare.^43^

Additionally, light-harvesting antenna complexes in bacteria and plants consist of various systematic porphyrin derivatives with visible photon absorption proficiency, resulting in charge separation and energy transfer relay in the reaction center.^44^ However, significant efforts are still needed to replicate such systems.^45^ Therefore, many systems were constructed including post synthetically anchoring photoactive porphyrin dye within the 1D pores of pyrene -derived NU-1000 MOF,^46^ installation of two electronically diverse carboxy-terminated zinc porphyrins into the hexagonal pores of NU-1000 *via* solvent-assisted ligand incorporation,^43^ porphyrin-based MOF incorporating C_60_,^47^ pyrene and porphyrin-based mixed-ligand metal–organic frameworks,^48^ mixed guest Ru(ii)(2,2′-bipyridine) : Mn(iii)tetrakis(4-sulphonatophenyl) porphyrine@HKUST-1 metal organic framework,^49^ fullerene loading in a porphyrin-based metal–organic framework through pore selectivity,^50^ and functionalization of a donor–acceptor mixed-ligand MOF to form a high-density donor–acceptor structure in an artificial porous light-harvesting system.^51,52^ However, the investigation of photoinduced electron and energy transfer through supramolecular systems constructed from non-covalently interacting chromophores and MOFs in aqueous environments remains limited besides the low internal energy transfer within the MOF to external energy acceptors.^41^

Herein, an MOF-based artificial light-harvesting system has been fabricated using a perlyene-based nickel MOF, namely TMPyP/Ni-PTCA-SDS nanosheets. The well-synthesized Ni-PTCA-SDS nanosheets contain individual perylene structural units that are tightly packed together through nickel metal, mimicking the sheet-like thylakoids in chloroplasts. These nanosheets, treated with sodium dodecyl sulfate to maintain electronegative active sites, facilitate efficient electrostatic interactions with electropositive TMPyP in well-organized and tunable TMPyP/Ni-PTCA-SDS self-assembly nanostructures. TMPyP is coupled with Ni-PTCA-SDS nanosheets, thereby constructing a TMPyP/Ni-PTCA-SDS supramolecular system in an aqueous medium that successfully converts solar energy into chemical energy with high solar spectrum absorption. Both the photophysical properties of the well-dispersed Ni-PTCA-SDS in water, the Förster resonance energy transfer (FRET), and the photoinduced charge transfer kinetics of the TMPyP/Ni-PTCA-SDS supramolecular system in water were examined using spectroscopic investigations and electrochemical studies, Fig. 1.

**Fig. 1:**
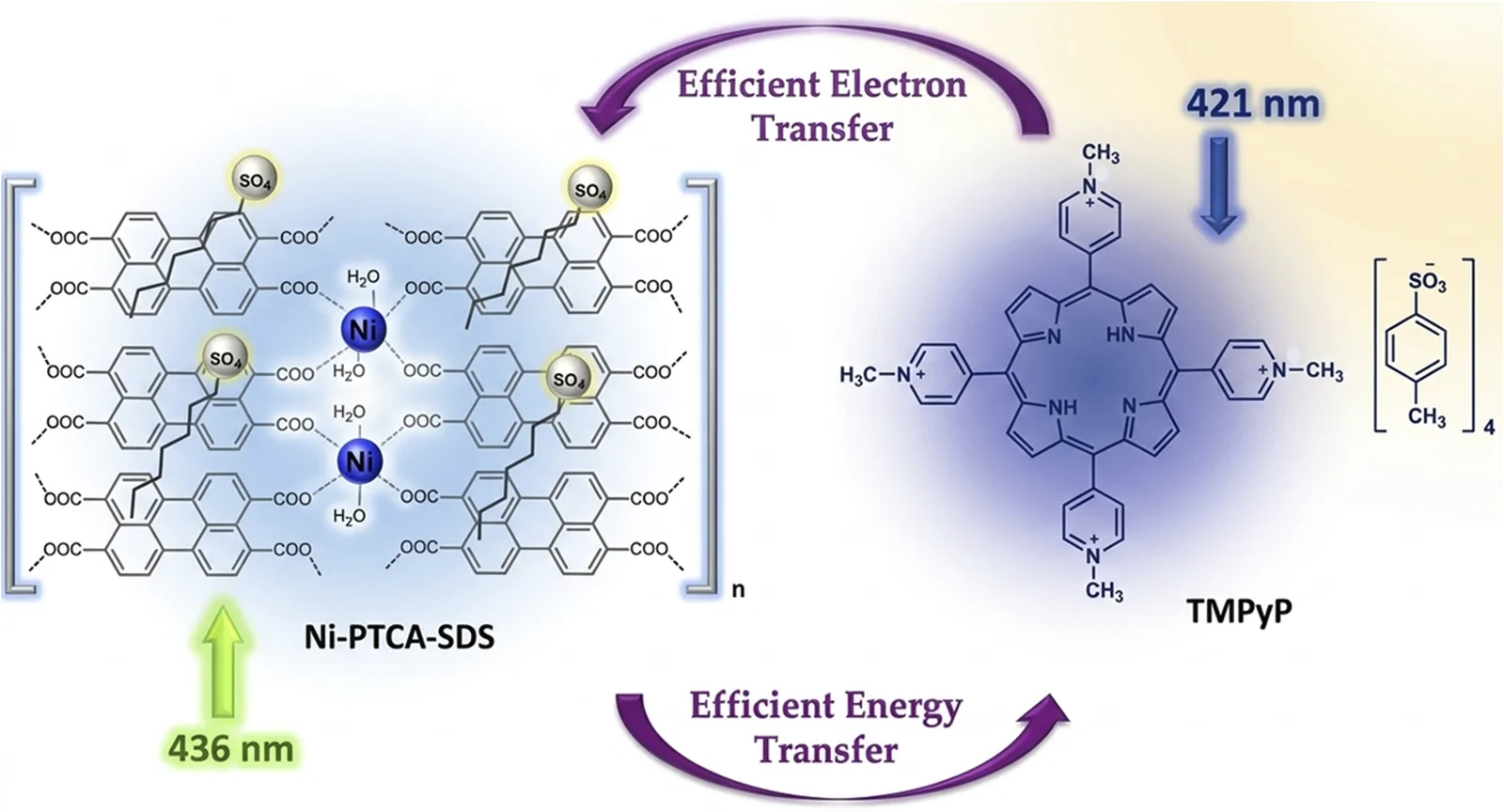
Schematic representation of the electrostatically driven self-assembly between TMPyP and Ni-PTCA-SDS.

## 2. Experimental section

### 2.1. Chemicals and materials

4,4′,4″,4‴-(Porphyrin-5,10,15,20-tetrayl)tetrakis(1-methylpyridin-1-ium) 4-methylbenzenesulfonate (TMPyP), nickel(ii) acetate tetrahydrate, sodium dodecyl sulfate, and perylene-3,4,9,10-tetracarboxylic dianhydride were purchased from Aldrich and utilized without any further purification.

### 2.2. Fabrication of Ni-PTCA MOF

Ni-PTCA MOF was synthesized according to the reported literature,^53^Fig. 2. Initially, perylene-3,4,9,10-tetracarboxylic dianhydride(PTCDA) was completely hydrolyzed by NaOH. 100 mg of PTCDA and 40 mg of sodium hydroxide are dispersed in 15.9 mL of deionized water. Then, the aqueous solution was stirred at 70 °C for 24 h. After PTCDA is completely hydrolyzed to form a fluorescent-green Na-PTCA aqueous solution, 15.9 mL of deionized water containing 179 mg of nickel acetate tetrahydrate was added under vigorous stirring. The aqueous solution is transferred to a polytetrafluoroethylene-lined reactor and reacted at 120 °C for 24 h. After natural cooling to room temperature, the Ni-PTCA MOF was washed with deionized water and ethanol and dried in a vacuum oven at 60 °C for 24 h.

**Fig. 2:**
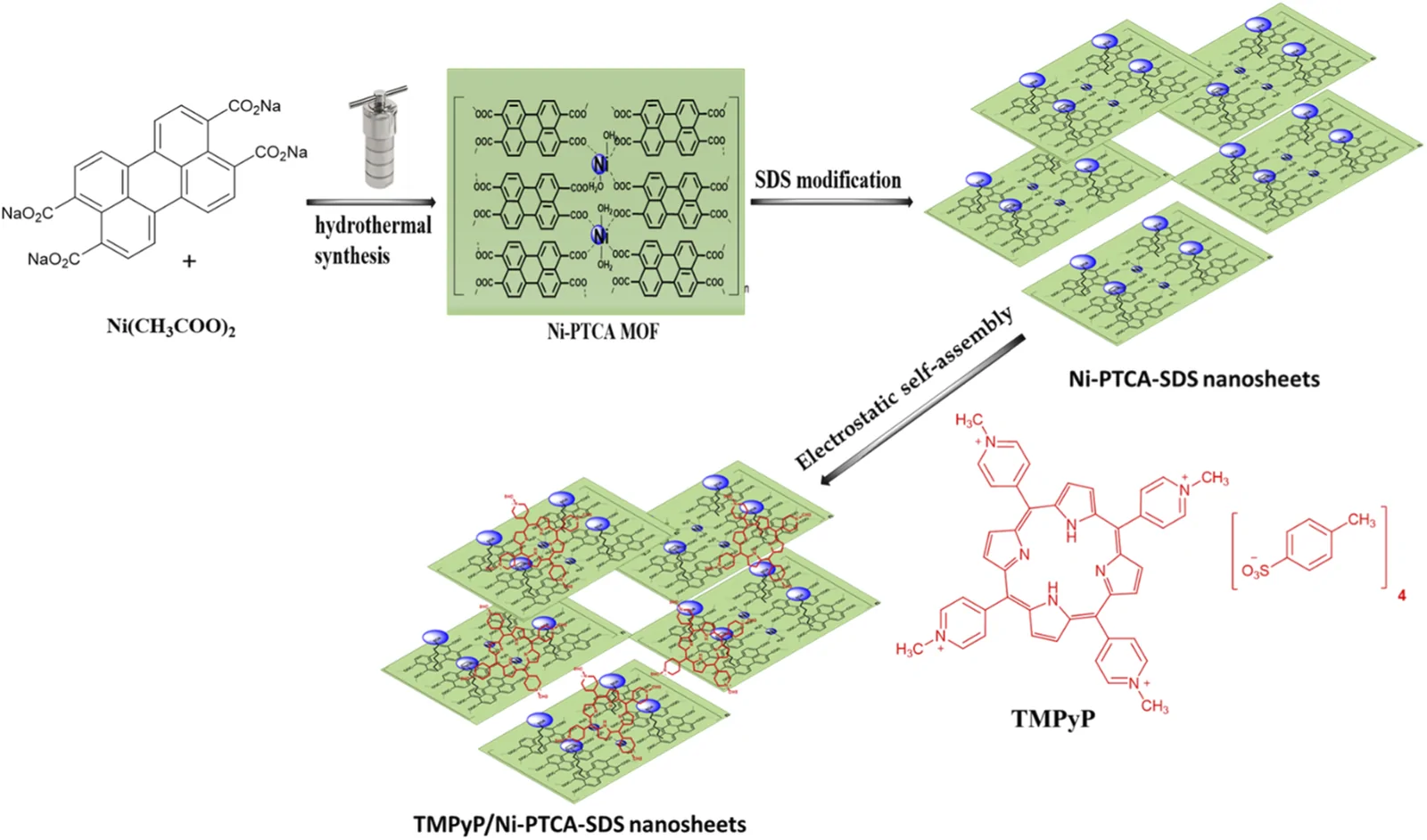
Schematic diagram for the fabrication of light-harvesting TMPyP/Ni-PTCA-SDS nanosheets.

### 2.3. Fabrication of Ni-PTCA-SDS nanosheets

2 mL of sodium dodecyl sulfate (SDS) aqueous solution (20 mg mL^−1^) was added to 8 mL Ni-PTCA MOF suspension (1.3 mg mL^−1^), then stirred at ambient temperature for 15 h. Finally, the crude product obtained was washed three times with ethanol and dried in a vacuum oven at 60 °C for 24 h.

### 2.4. Fabrication of the TMPyP/Ni-PTCA-SDS 2D nanosheets

8 mL Ni-PTCA-SDS suspension (30 mg) are mixed with 2 mL of porphyrin derivative aqueous solution (1 mg mL^−1^) and stirred at ambient temperature for 24 h. The resulting mixture was filtered, and the solid product was washed twice with water, and dried for 24 h in a vacuum oven at 60 °C. The fabrication method is summarized in Fig. 2.

### 2.5. Instruments

Crystallographic structures of the synthesized materials were confirmed by XRD (Shimadzu-X-Lab 600, Japan), which was performed at 40 kV with Cu Kα radiation. Functional groups of the synthesized materials were detected using Attenuated Total Reflection Fourier Transform Infrared ATR-FTIR by Bruker INVENIO-S. The infrared spectrometer (Bruker Alpha Platinum, Germany) with an internal reflection element (diamond) with a resolution of 4 cm^−1^ and a sweep of 32 scans in the mid-infrared range (4000–500 cm^−1^) was used. A zeta potential analyzer from Malvern Nano ZS Instruments was utilized to examine the surface charge. K-Alpha X-ray Photoelectron Spectroscopy (XPS) Thermo Fisher Scientific-US was applied to confirm the surface chemistry, elemental composition, and bonding status. The surface morphology was investigated using a scanning electron microscope (SEM), a JEOL (JSMIT100) 30 kV, and a laser microscope. The high-resolution transmission electron microscopy (HR-TEM) technique (TEM model JEOL JEM-2100F, Japan) at 200 kV was applied to visualize the morphologies of the fabricated materials. Steady-state absorption and fluorescence measurements were obtained using a UV-vis spectrophotometer (Shimadzu UV-2600) and spectrofluorometer (Shimadzu RF-6000), respectively. The picosecond fluorescence decay profiles of synthesized materials were investigated by the single photon counting technique utilizing FluorHub (Horiba Scientific). Lifetime detected from the software Decay Analysis 64 by Horiba Scientific, linked to the equipment.

Photoelectrochemical measurements were performed using a 300 W Xenon arc lamp equipped with an optical cut-off filter (*λ* ≥ 420 nm) and potentiostat workstation (CorrTest Instruments, model CS350). Ag/AgCl as a reference electrode, Pt wire as a counter electrode, and FTO (Fluorine doped tin oxide) coated with the composite paste as a working electrode are used. All electrodes were soaked in Na_2_SO_4_ electrolyte (0.1 M), filling the electrochemical cell. To prepare the working electrode, 20 mg of each powder was mixed with 1 mL of isopropanol. The FTO substrate (1.5 cm × 1 cm) was thoroughly cleaned in deionized water and isopropanol through multiple sonication cycles. Subsequently, 200 µL of the solution was dropped-casted onto the substrate in 50 µL increments. After finishing, the electrode was kept drying in room temperature. Before the investigation, the electrolyte was purged with Ar gas and irradiated with light to initiate the photo-electroreaction. The applied electrochemical tests were: (a) cyclic voltammetry (CV) with a potential window of 1 to −1 V *vs.* Ag/AgCl, Sat. KCl and a scan rate of 50 mV s^−1^, (b) electrochemical impedance spectroscopy (EIS) conducted within a frequency range of 10^5^ to 0.01 Hz, with a 20 mV amplitude signal, (c) chronoamperometry photocurrent response (CA) performed for 660 s at a potential of −0.8 and +1.0 V to determine the photocurrent response.

The fluorescence quantum yield of the Ni-PTCA-SDS material was determined relative to *N*,*N*′-bis(2-(trimethylammoniumiodide) ethylene) perylenediimide (TAIPDI) as a reference (*Φ*_f_ = 0.11 in water) according to eqn (1).^54^1
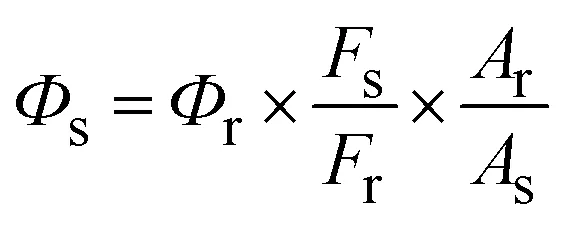
where *Φ*_s_ and *Φ*_r_ refer to the sample's and reference's quantum yields, respectively. *F*_s_ and *F*_r_ are the integrated fluorescence intensity of the sample and reference, respectively. *A*_r_ and *A*_s_ are the optical densities of the reference and sample, respectively, measured at 436 nm.

## 3. Results and discussion

### 3.1. Structural characterization of Ni-PTCA MOF, Ni-PTCA-SDS nanosheets and 2D TMPyP/Ni-PTCA-SDS nanosheets

The FTIR spectrum of Ni-PTCA MOF exhibited characteristic peaks at 1547, and 1590 cm^−1^, pointing to –COO^−^Ni^2+^ symmetric stretching vibration, confirming the coordination between PTCA ligand and Ni^2+^ centers, Fig. 3a. Additional bands observed at 460 and 809 cm^−1^ are assigned to out-of-plane bending vibration of perylene and out-of-plane twisting vibration of C–H, respectively. Upon SDS modification, the FTIR spectrum of the Ni-PTCA-SDS nanosheets, Fig. 3a, retains all characteristic vibrational bands of the parent Ni-PTCA MOF, particularly the carboxylate–metal coordination bands, indicating that the incorporation process does not disrupt the intrinsic coordination environment or chemical framework of the MOF. This confirms the formation of a structurally stable hybrid composite. On the other hand, incorporation of SDS into Ni-PTCA MOF is evidenced by characteristic peaks at 2848 and 2913 cm^−1^ resembling distinct C–H stretching modes of the aliphatic hydrocarbon chain of SDS. Furthermore, TMPyP/Ni-PTCA-SDS composite exhibited the same diagnostic features of Ni-PTCA-SDS FTIR spectrum, proving the construction of a stable composite with a well-maintained chemical structure of MOF, Fig. 3a. Additionally, a characteristic peak at 1638 is recorded, which is assigned to the imine group (C=N), confirming the presence of TMPyP and its combination with Ni-PTCA-SDS in TMPyP/Ni-PTCA-SDS nanosheets.

**Fig. 3:**
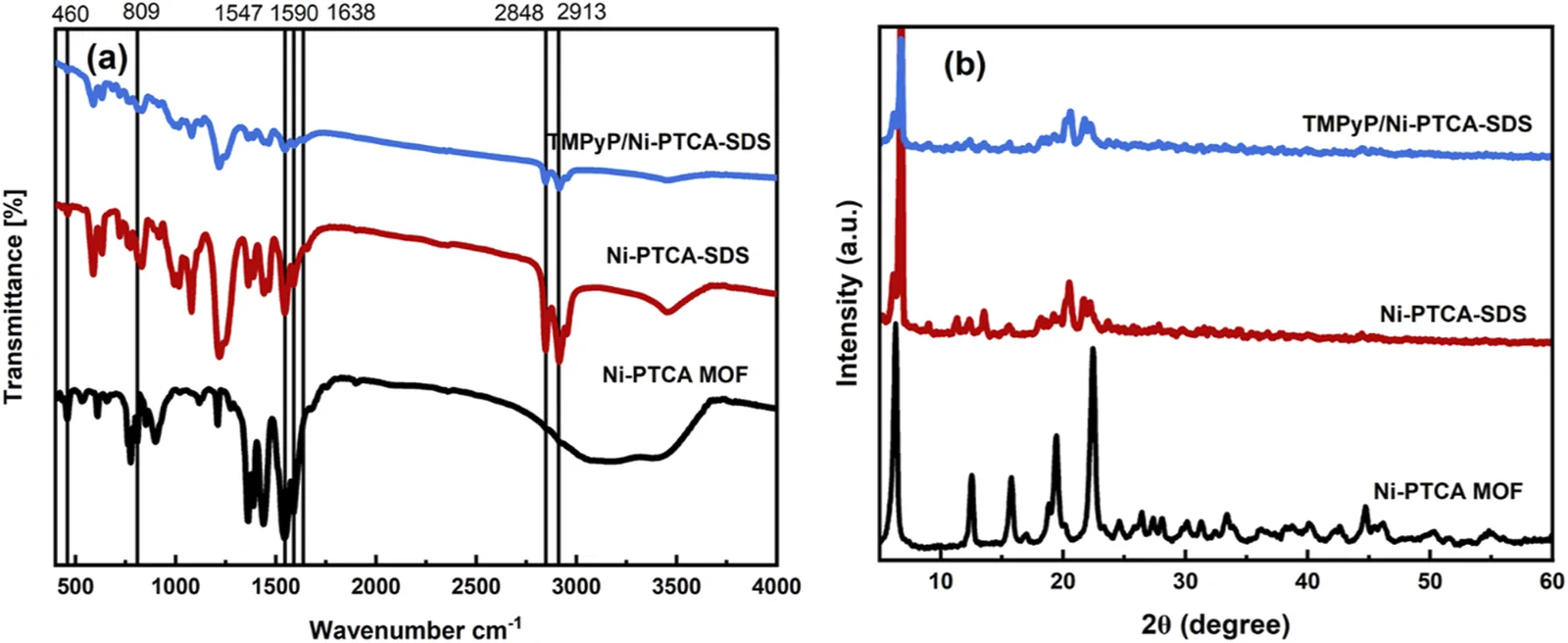
Structure analysis of Ni-PTCA MOF, Ni-PTCA-SDS, and TMPyP/Ni-PTCA-SDS; (a) FTIR spectra, (b) X-ray diffraction patterns measurements.

Powder X-ray diffraction (PXRD) patterns of the Ni-PTCA MOF material are in good agreement with the predicted patterns from the literature^54^ and prove the formation of a crystalline Ni-perlyene framework, Fig. 3b. X-ray diffraction (XRD) analysis of Ni-PTCA MOF showed a dominant low-angle reflection at 6.27° (*d* = 1.40 nm) corresponding to the crystal face of (001) and resembling the long-range periodicity of the MOF structure and evidence of overlapped coordination structures. A characteristic peak at 12.50° (*d* = 0.70 nm), 15.73° (*d* = 0.56 nm), 19.41° (*d* = 0.45 nm), and 22.40° (*d* = 0.39 nm) can be attributed to the distance between the adjacent ordered π–π stacking perylene molecules and that between Ni–carboxylate connectivity, corresponding to crystal faces of (002), (110), (201), and (202), respectively.^54^

Furthermore, the characteristic peaks of Ni-PTCA MOF are retained with noticeable peak shift and broadening upon treatment with SDS, assigned to the successful adsorption of SDS on Ni-PTCA MOF framework, causing directional changes in interplanar spacing while maintaining underlying metal–ligand connectivity and crystalline framework. The Ni-PTCA-SDS composite exhibited intense low angle reflection compared to that of Ni-PTCA MOF, indicating of enhancement of long-range order, Fig. 3b. The observations of XRD measurement of SDS effect on MOF are consistent with previously reported literature^55–58^ and correlate with change of shape and size in DLS, SEM, and HR-TEM measurements. The PXRD patterns of TMPyP/Ni-PTCA-SDS nanosheets were compatible with those of Ni-PTCA-SDS material, elucidating no structural disruption by surface interaction. The X-ray photoelectron spectroscopy (XPS) analysis was performed to further examine the bonding states and surface chemistry of Ni-PTCA MOF (Fig. S1). The XPS of O 1s consisted of carboxyl oxygen at 531.8 eV and weakly coordinated oxygen at 533.6 eV. Meanwhile, the O 1s XPS spectrum of the Ni-PTCA-SDS and TMPyP/Ni-PTCA-SDS exhibits two peaks at 531.2 and ∼533 eV, indicating that the Ni-PTCA MOF framework remains structurally intact, and that SDS modification occurs without decomposition of the MOF (Fig. S2 and S3). The Ni 2p XPS spectrum of both Ni-PTCA MOF, Ni-PTCA-SDS, and TMPyP/Ni-PTCA-SDS displays characteristic Ni^2+^ features with a Ni 2p_3/2_ peak at 856 eV and a Ni 2p_1/2_ peak at 873 eV, accompanied by pronounced shake-up satellite structures, indicative of Ni^2+^ species coordinated with oxygen in both frameworks.

The zeta potential measurements were performed to give insight into the surface charge and stability of the investigated materials. Ni-PTCA MOF has a zeta potential of −27.6 mV, reflecting the low colloidal stability of the negatively charged surface MOF. Conversely, the zeta potential of Ni-PTCA-SDS nanosheets displayed −42.5 mV, confirming the key role of SDS incorporation in Ni-PTCA MOF crystal by increasing the stability of Ni-PTCA-SDS nanosheets and achieving electrostatic interaction with TMPyP dye in water. Being higher than that of Ni-PTCA-SDS, the negatively charged surface of TMPyP/Ni-PTCA-SDS composite with a zeta potential of −39.9 mV proved the adsorption of positively charged TMPyP on Ni-PTCA-SDS nanosheets *via* electrostatic interaction and possesses a convenient stability of the composite.

Dynamic light scattering (DLS) analysis was performed for a general hydrodynamic size estimation, Fig. S4. The particle hydrodynamic size of Ni-PTCA MOF was determined using dynamic light scattering analysis (DLS) to be 1.7 µm, Fig. S4, aligned with other reported values of MOF particle size.^59^ The Ni-PTCA-SDS nanosheets have apparent hydrodynamic size of 0.9 µm, illustrating the incorporation of SDS into MOF that causes a decrease in size compared with that of Ni-PTCA MOF. According to the previously reported impact of SDS,^60^ this decline in hydrodynamic size can be attributed to electrostatic repulsion of negatively charged sulfate groups of SDS, leading to exfoliation of layered Ni-PTCA MOF into two-dimensional nanosheets, which is confirmed by SEM and HR-TEM analysis of Ni-PTCA-SDS. Whereas the measurable increase in apparent hydrodynamic size of TMPyP/Ni-PTCA-SDS composite (1.1 µm) accounts for the formation of TMPyP/Ni-PTCA-SDS composite through electrostatic self-assembly interaction.

### 3.2. Morphological characterization

The scanning electron microscopy (SEM) characterization revealed the formation of Ni-PTCA MOF, which is consistent with the reported literature.^54^ An open framework with a three-dimensional wavy-layered structure of Ni-PTCA MOF was outlined in the SEM image, Fig. 4a–c, resulting from the six-coordination of the central Ni^2+^ ion with the organic ligands in an octahedral symmetry, which consists mainly of four-coordination with PTCA and is intertwined by H_2_O, leading to planar disruption of perylene units. Moreover, the impact of SDS on Ni-PTCA MOF material was macroscopically clarified by the remarkable change of morphology in SEM analysis of Ni-PTCA-SDS. The SEM image of Ni-PTCA-SDS MOF in Fig. 4d–f shows an obvious two-dimensional nanosheet morphology. The SEM images, as illustrated in Fig. 4g–i, showed that the TMPyP/Ni-PTCA-SDS nanosheets with more intense dark regions were successfully fabricated, confirming the effective deposition of TMPyP onto the Ni-PTCA-SDS nanosheets through electrostatic interactions.

**Fig. 4:**
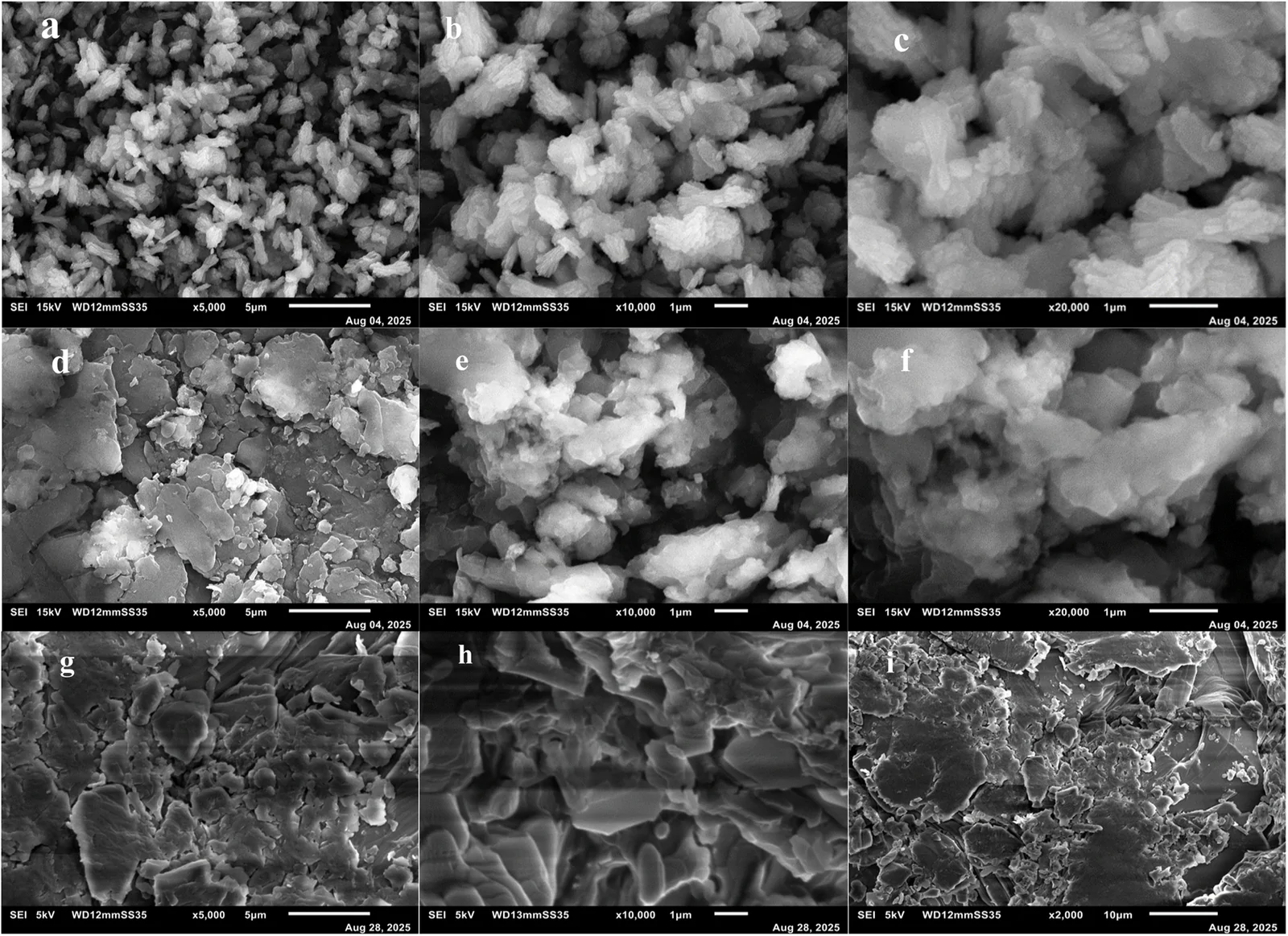
Scanning electron micrograph (SEM) images of Ni-PTCA MOF (a–c), Ni-PTCA-SDS (d–f), and TMPyP/Ni-PTCA-SDS (g–i).

More detailed visualization of the synthesized materials' morphology was investigated using high-resolution transmission electron microscopy at varied magnifications. HR-TEM images of Ni-PTCA MOF reveal the formation of a three-dimensional wavy-layered sheet-like structure in accordance with SEM images, as shown in Fig. 5a and b. The magnified TEM images of Ni-PTCA-SDS confirm the formation of a 2D nanosheet array with relatively high contrast, which can be attributed to the stacking of layers and the possibility of increased electron scattering from metal, as detected by the SEM analysis, Fig. 5c and d. In addition, the HR-TEM images of TMPyP/Ni-PTCA-SDS composite, as illustrated in Fig. 5e and f, showed the construction of a uniform, flat, and stacked together nanosheet morphology, confirming the electrostatic adsorption of TMPyP on Ni-PTCA-SDS without any changes to its morphology, as confirmed with XRD analyses.

**Fig. 5:**
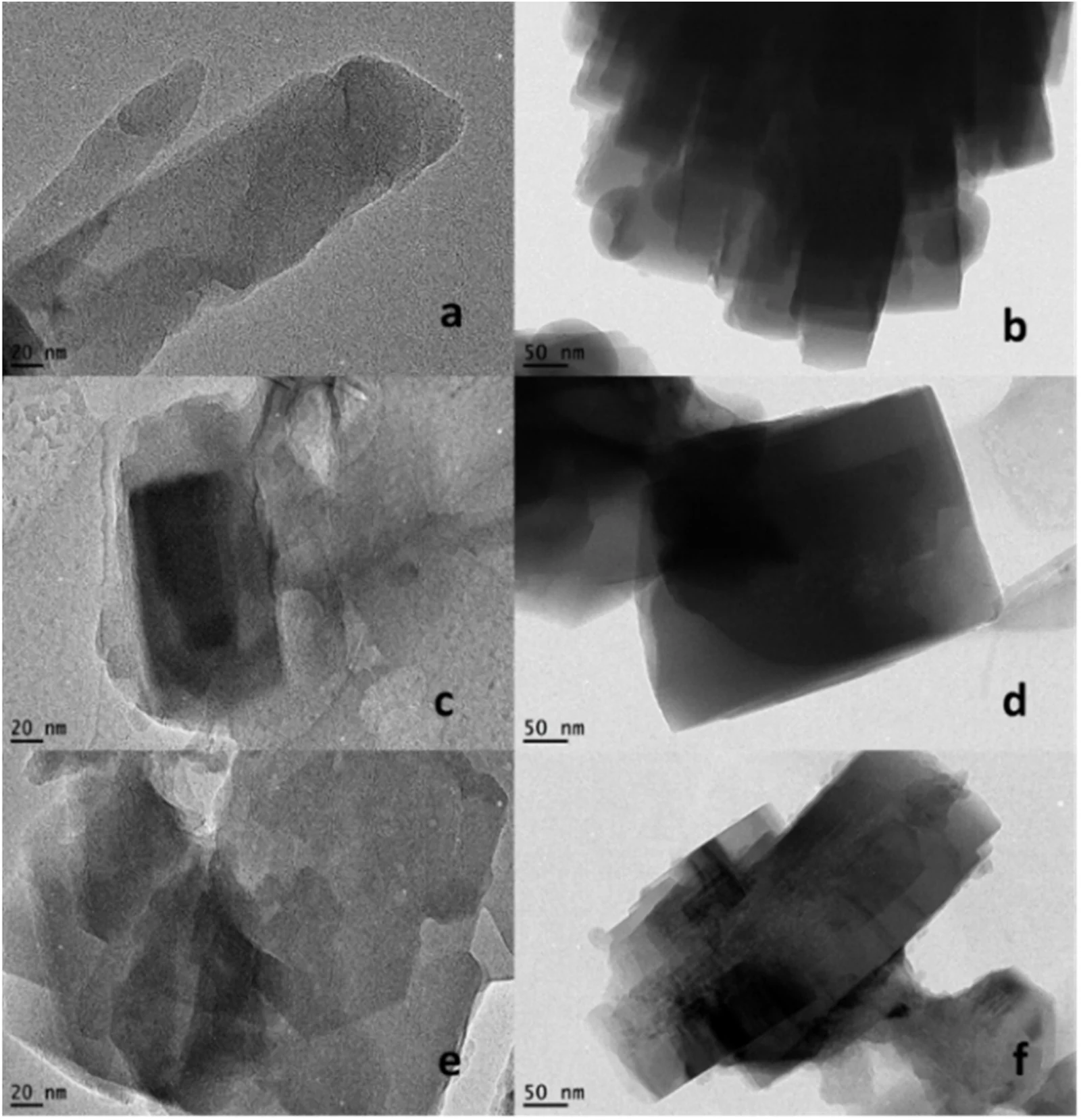
HR-TEM images of Ni-PTCA MOF (a and b), Ni-PTCA-SDS (c and d), and TMPyP/Ni-PTCA-SDS (e and f) at 20 nm and 50 nm magnification degrees.

As depicted in Fig. 6a, the energy-dispersive X-ray (EDX) mapping of Ni-PTCA MOF confirmed the uniform distribution of C, O, and Ni elements within the material. Interestingly, elemental mapping of Ni-PTCA-SDS reveals a homogeneous distribution of sulfur throughout nanosheets, supporting the integration of SDS molecules with Ni-PTCA MOF framework and adsorption on MOF surface, Fig. 6b. The uniform distribution of nitrogen is shown in the EDX mapping of TMPyP/Ni-PTCA-SDS composite, enhancing the electrostatic interaction of TMPyP compound with Ni-PTCA-SDS, Fig. 6c.

**Fig. 6:**
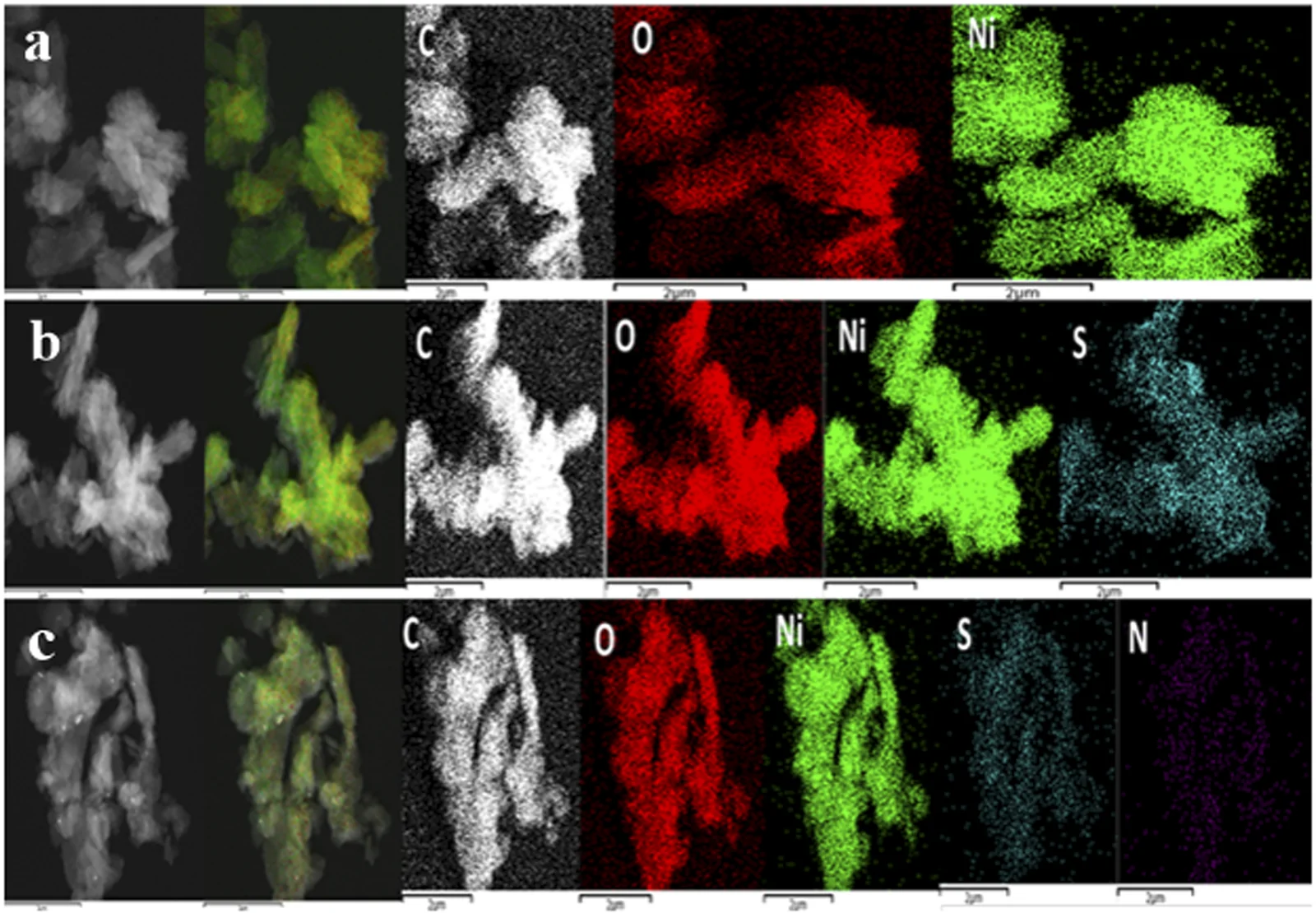
EDX mapping images of: (a) Ni-PTCA MOF, (b) Ni-PTCA-SDS, (c) and TMPyP/Ni-PTCA-SDS.

### 3.3. Optical studies

The UV-vis absorption spectra of TMPyP in water showed a distinct Soret band at 421 nm, as well as four weak *Q* vibronic bands at 517, 554, 585, and 648 nm, as indicated in Fig. 7d. It possesses a strong emission band at 711 nm on excitation at 421 nm. In addition, the absorption spectrum of the sodium salt of perylene-3,4,9,10-tetracarboxylate in water shows three pronounced peaks at 412, 436, and 464 nm, Fig. 7a. Compared to the sodium salt of PTCA, the well-dispersed Ni-PTCA-SDS nanosheets in water display absorption across the entire full solar spectrum, with characteristic absorption peaks assigned to the perylene-3,4,9,10-tetracarboxylate core unit, Fig. 7a. The absorption spectrum of Ni-PTCA-SDS nanosheets in water showed a broad absorption maximum at 436 and 464 nm, confirming the presence of perylene tetra carboxylate unit in Ni-PTCA-SDS nanosheets, and significant absorption of the 500–800 region, proving the metal to ligand charge transfer interaction between nickel metal and perylene-3,4,9,10-tetracarboxylate core unit and d–d transition. On the other hand, the fluorescence spectrum of the sodium salt of PTCA offered two strong emission bands at 480 and 510 nm and a shoulder at 554 nm upon irradiation of its aqueous solution at 436 nm, Fig. 7b.

**Fig. 7:**
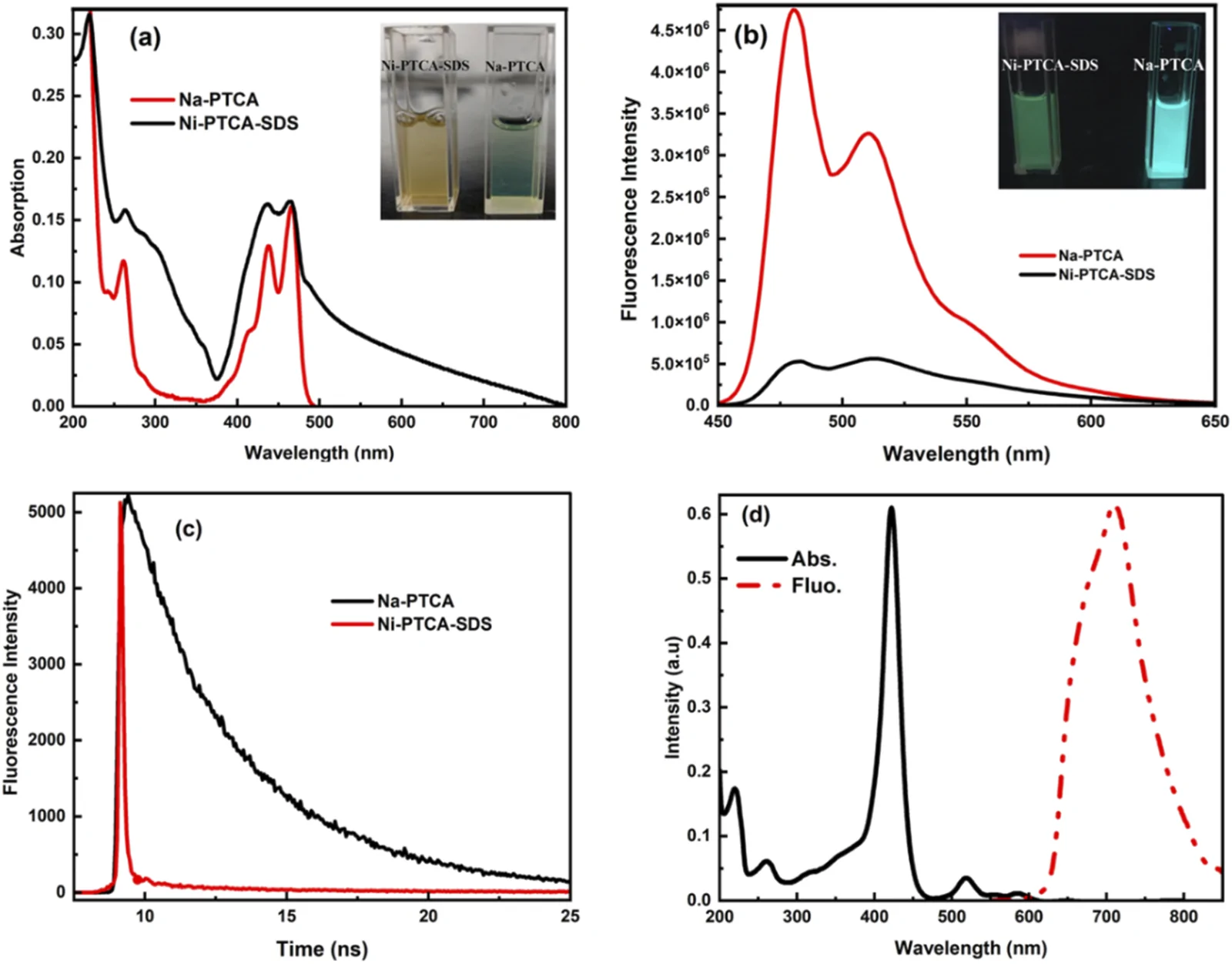
(a) Absorption spectra of Na-PTCA and Ni-PTCA-SDS in water. (b) Fluorescence spectra of Na-PTCA and Ni-PTCA-SDS nanosheets in water *h*_ex_ = 436 nm. (c) Corresponding fluorescence decay traces of both singlet Na-PTCA and Ni-PTCA-SDS in water (*λ*_ex_ = 436 nm, *λ*_em_ = 480 nm). (d) Normalized absorption and fluorescence spectra of TMPyP, *λ*_ex_ = 421 nm.

Whereas the emission spectra of Ni-PTCA-SDS solution was heavily quenched compared with that of Na-PTCA upon excitation at the same wavelength, which is assigned to ligand to metal charge transfer,^54,61,62^ as well as evidencing the formation of Ni-PTCA-SDS 2D nanosheets. The fluorescence spectrum of Ni-PTCA-SDS exhibited two emission bands and a shoulder with a slight red shift (2–4 nm) compared with that of the sodium salt of PTCA, at 482, 513, and 558 nm, Fig. 7b. Fluorescence lifetime measurements of the examined materials coincide with the steady-state fluorescence measurements. As shown in Fig. 7c, the time profile of the singlet excited state of Na-PTCA displayed monoexponentially decay with a lifetime of 4.12 ns. In contrast, the fluorescence decay profile of the Ni-PTCA-SDS nanosheets exhibited biexponential behavior, which is markedly shorter than that of Na-PTCA. The fast decay component (<50 ps, beyond the sensitivity limit of our TCSPC setup) reflects charge transfer between the nickel center and the perylene-3,4,9,10-tetracarboxylate core, while the slow component (3.08 ns) corresponds to the decay of the perylene core itself.^54,61,62^

### 3.4. Self-assembly for TMPyP/Ni-PTCA-SDS nanosheets and photophysical studies

Upon photometric titration of TMPyP aqueous solution with different concentrations of Ni-PTCA MOF, no substantial change in the absorption spectra of TMPyP could be recorded, as illustrated in Fig. S5. Similarly, fluorescence intensity of the singlet-excited state related to TMPyP revealed no considerable change upon complexation with different amounts of Ni-PTCA MOF. Based on absorption and fluorescence measurements, complex formation between TMPyP and Ni-PTCA MOF could not be constructed. To efficiently integrate the Ni-PTCA MOF and TMPyP together, the electrostatic self-assembly strategy was applied. The SDS-modified Ni-PTCA MOF (Ni-PTCA-SDS), possessing strong electronegativity features, efficiently adsorbed the electropositive TMPyP. Optical studies of the TMPyP/Ni-PTCA-SDS nanosheets combination showed significantly different features compared to those of TMPyP/Ni-PTCA MOF. Titration of the constant concentration of TMPyP aqueous solution with increasing concentration of Ni-PTCA-SDS aqueous solution elucidates an obvious decline of the Soret band at 420 nm, accompanied by a notable red shift (7 nm), and the appearance of two distinct isosbestic points at 334 and 439 nm, Fig. 8a. This observed red shift in the porphyrin Soret band is comparable to those reported for supramolecular donor–acceptor light-harvesting assemblies.^12,14,54^

**Fig. 8:**
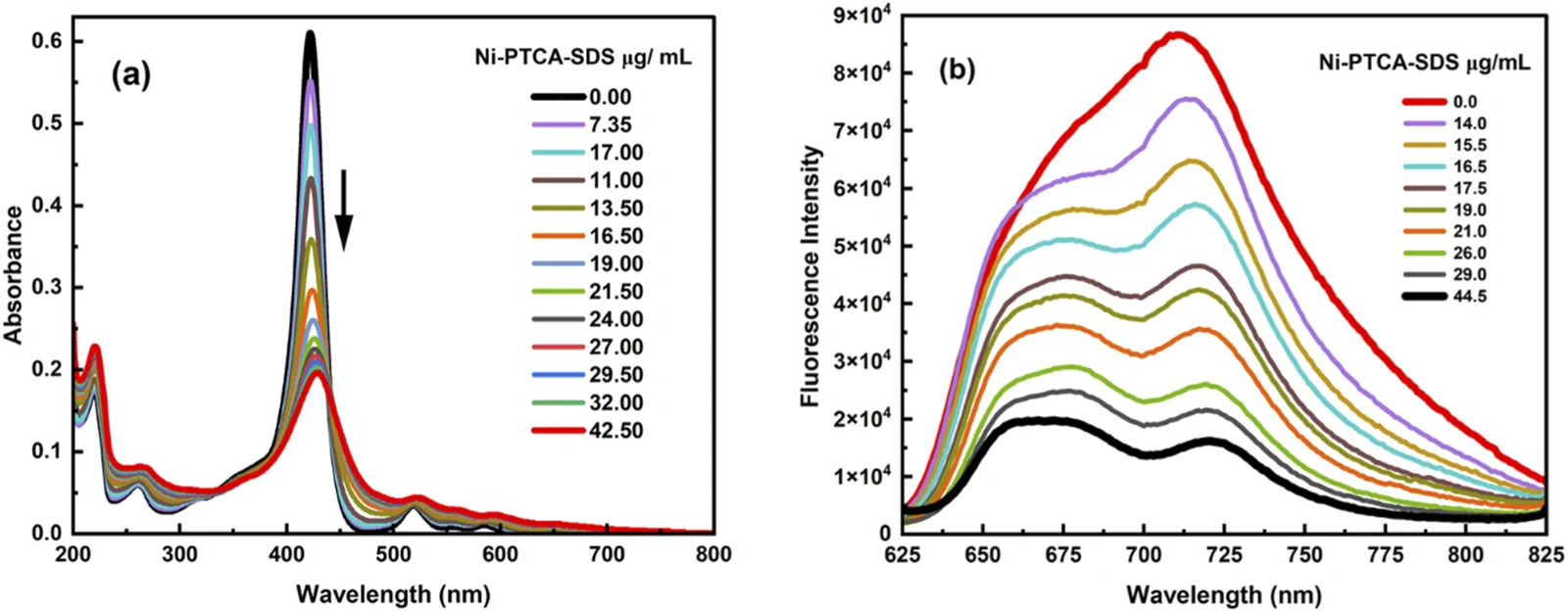
(a) Absorption spectral mutations of TMPyP (16.4 × 10^−4^ mM) during the addition of various Ni-PTCA-SDS concentrations. (b) Fluorescence spectral effect of Ni-PTCA-SDS addition on TMPyP (16.4 µM) emission spectra.

Moreover, absorption of four weak *Q* vibronic bands at 517, 554, 585 and 648 nm was enhanced with a slight red shift to be 522, 560, 598, and 656 nm, Fig. S6. This behavior can be attributed to the growth of Ni-PTCA-SDS absorption parallel to the electrostatic interaction with the TMPyP. These observations affirm the construction of a supramolecular complex between TMPyP and Ni-PTCA-SDS nanosheets in aqueous solution *via* electrostatic self-assembly. The loading efficiency of TMPyP on the Ni-PTCA-SDS framework was calculated by tracking the absorption decline of a known initial TMPyP concentration in the supernatant to be 0.69%, Fig. S7.

Utilizing steady-state fluorescence spectroscopy, the formation of TMPyP/Ni-PTCA-SDS supramolecular light-harvesting system was evidenced. As depicted from the fluorescence spectrum in Fig. 8b, TMPyP displays a strong emission band at 711 nm upon excitation of its aqueous solution at 421 nm; meanwhile, gradual addition of Ni-PTCA-SDS to the TMPyP in water illustrates significant fluorescence intensity quenching of the singlet-excited state of TMPyP.

Notably, the fluorescence emission of TMPyP exhibits a concentration-dependent response upon the addition of SDS below its critical micelle concentration (CMC), characterized by an initial quenching followed by a gradual recovery and enhancement of emission intensity at higher SDS concentrations, Fig. S8. This behavior is attributed to dye disaggregation and improved solvation induced by excess SDS. These observations are consistent with previous reports and support the formation of the TMPyP/Ni-PTCA-SDS supramolecular system.^63,64^ Furthermore, two-dimensional (2D) and three-dimensional (3D) excitation–emission fluorescence maps were recorded to monitor changes in the emission behavior of TMPyP due to the Ni-PTCA-SDS interaction. The TMPyP exhibits a well-defined emission maximum centered at 711 nm upon excitation at 421 nm, as clearly visualized in both the 2D contour and 3D surface plots, Fig. 9a and b.

**Fig. 9:**
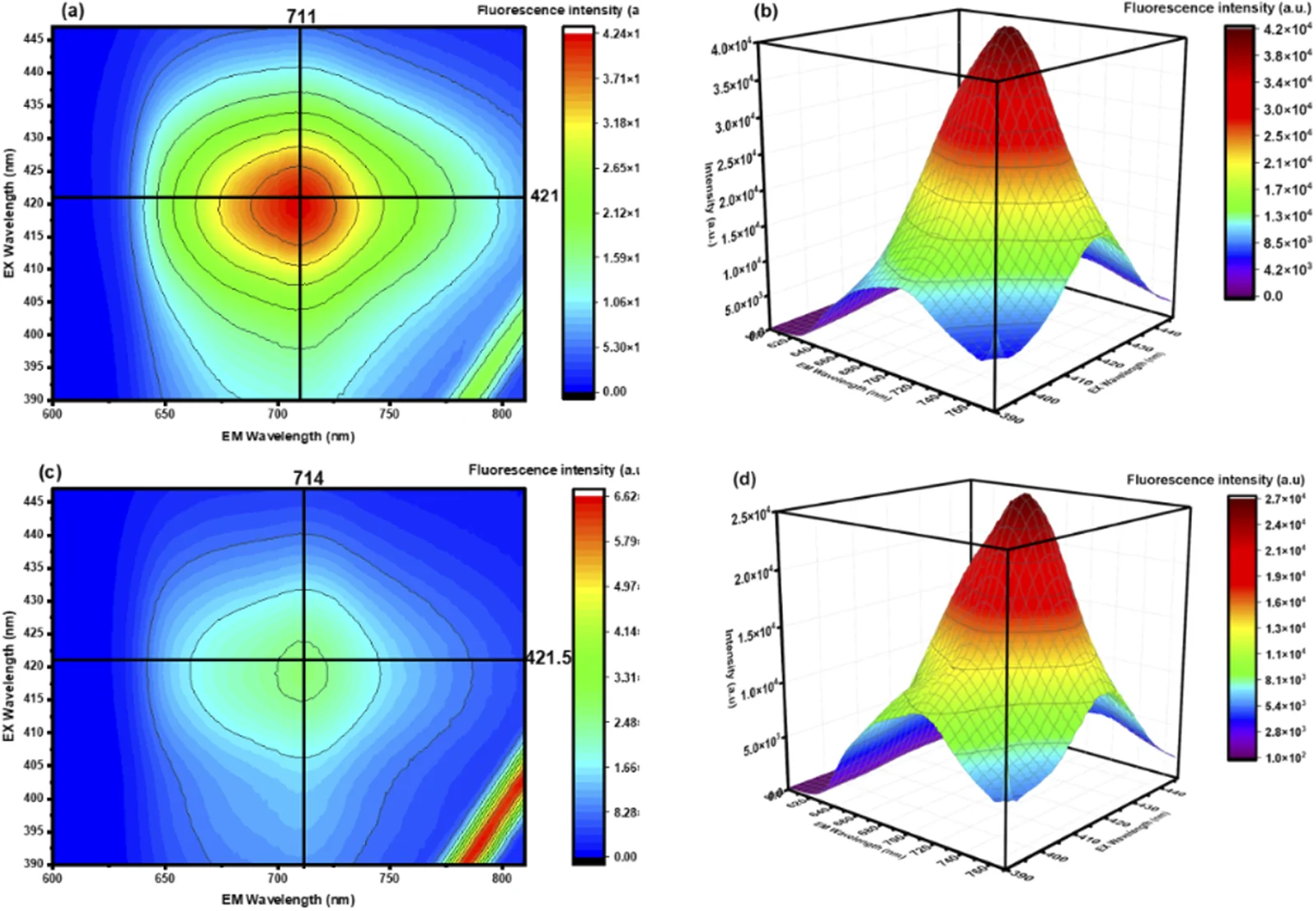
(a) Two-dimensional (2D) excitation–emission plot showing the relationship between excitation wavelengths (390–447 nm) and emission wavelengths of TMPyP. (b) 3D fluorescence surface map of TMPyP. (c) 2D excitation–emission plot illustrating the relationship between excitation wavelengths (390–447 nm) and emission wavelengths of the TMPyP/Ni-PTCA-SDS. (d) 3D fluorescence surface map of the TMPyP/Ni-PTCA-SDS.

Upon addition of Ni-PTCA-SDS (16 µg mL^−1^), the fluorescence intensity of TMPyP was significantly quenched with a slight red shift of maximum emission and excitation wavelengths to 714 and 421.5 nm, respectively, Fig. 9c and d. These findings indicate a fast and efficient intra-supramolecular electron transfer process from the electron-donating TMPyP to the electron-accepting Ni-PTCA-SDS of the electrostatic self-assembled TMPyP/Ni-PTCA-SDS composite.

Surprisingly, the incremental addition of TMPyP to an aqueous solution of dispersed nanosheets caused a considerable decrease in fluorescence emission of Ni-PTCA-SDS upon excitation of the composite moiety at 436 nm in conjunction with an increase of two growing emission bands centered at 676, 720 nm which are assigned to TMPyP emission, as seen in Fig. 10a. This behavior confirms the Förster resonance energy transfer (FRET), from energy donor Ni-PTCA-SDS nanosheets (2.57 eV) to the energy acceptor TMPyP (1.74 eV), originating from spectral overlap between Ni-PTCA-SDS emission and TMPyP absorption spectra, Fig. S9, with spectral overlap integral (*J* = 5.88 × 10^14^ M^−1^ cm^−1^ nm),^4^ which is calculated using eqn (2).

2 *J* = ∫*F*(*λ*)*ε*(*λ*)*λ*^4^Δ*λ*/∫*F*(*λ*)Δ*λ*

where *F*(*λ*) is the fluorescence intensity of energy donor Ni-PTCA-SDS at wavelength *λ*, while *ε*(*λ*) signifies the molar extinction coefficient of the energy acceptor TMPyP in M^−1^ cm^−1^ at wavelength *λ*. The Stern–Volmer plot for the singlet excited state quenching of Ni-PTCA-SDS aqueous solution by TMPyP exhibits a steep linear slope, indicating highly efficient quenching through electrostatic self-assembly interaction, Fig. 10b. The Stern–Volmer constant (*K*_sv_) was determined to be 2.8 × 10^5^ M^−1^. Moreover, FRET efficiency (*E*) is used to quantitatively estimate the efficiency of a light-harvesting system. Based on the fluorescence intensities of the donor measured in the absence (*F*_D_) and in the presence (*F*_DA_) of the acceptor, in addition to donor–acceptor separation distance (*r*) and Förster distance (*R*_0_), the energy transfer efficiency can be calculated according to the following eqn (3) and (4):3
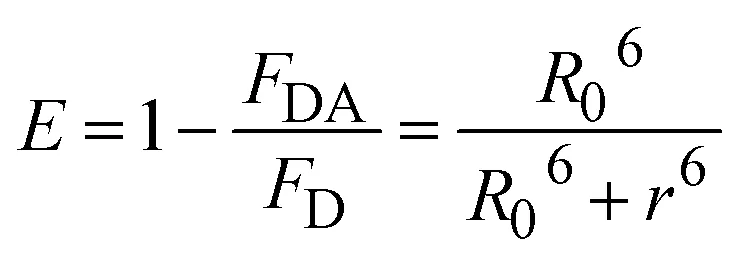

4 *R*_0_^6^ = 8.8 × 10^−25^*k*^2^*N*^−4^*ΦJ*

where *N* is the refractive index of water (1.333), *K* is the orientation factor of the dipole (*k*^2^ = 2/3), and *Φ* is the fluorescence quantum yield of the Ni-PTCA-SDS, estimated to be 0.06, Fig. S10. When the doping concentration of TMPyP is 60.7 × 10^−4^ mM, the FRET efficiency was calculated to be 69%. The separation distance between TMPyP and Ni-PTCA-SDS is equal to 3.6 nm, while the Förster distance (*R*_0_) for 50% energy transfer was determined to be 4.1 nm.

**Fig. 10:**
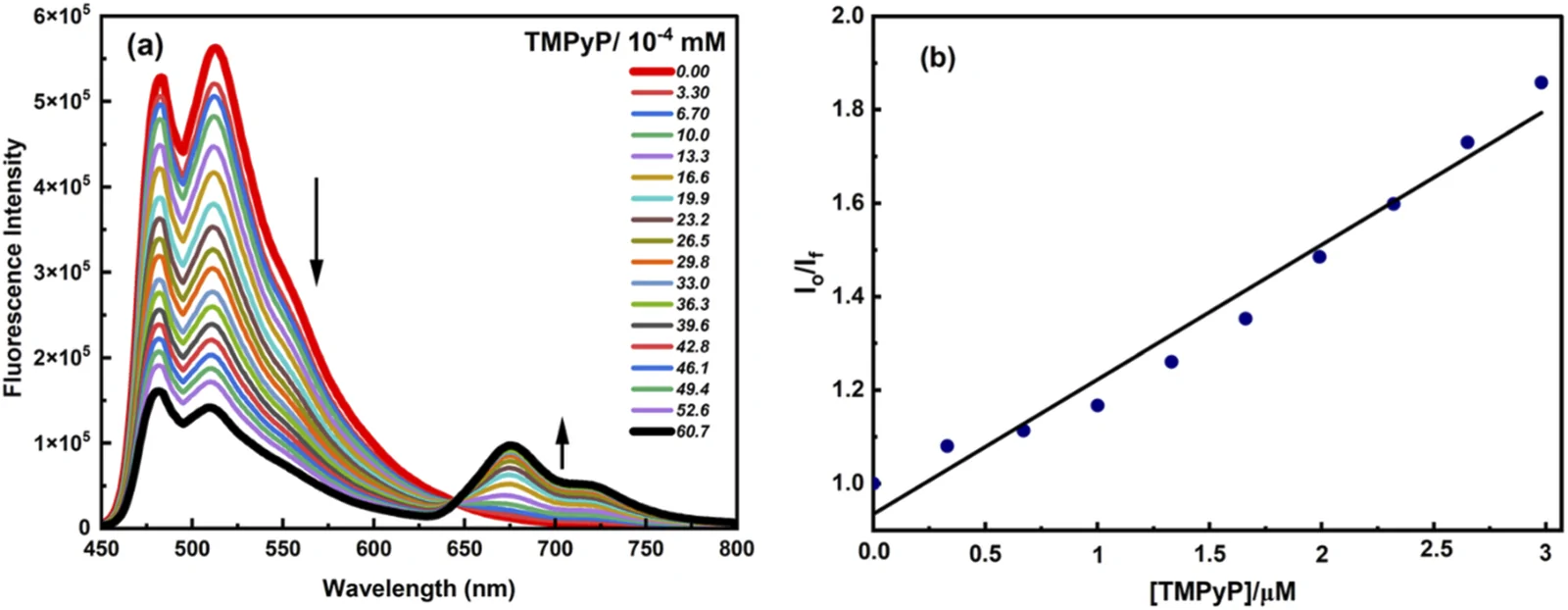
(a) Fluorescence spectral changes of Ni-PTCA-SDS (0.4 mg mL^−1^) upon progressive increase of TMPyP concentrations (0–14.7 µM), (b) Stern–Volmer plot for the fluorescence quenching of Ni-PTCA-SDS in the presence of different amounts of TMPyP in water; *λ*_ex_ = 436 nm.

Steady-state fluorescence inferences were quantitatively confirmed through the fluorescence lifetime measurements, yielding kinetics of the electron transfer features in water. Upon light irradiation at 421 nm, the fluorescence decay profile of the TMPyP single-excited state in water, detected at 711 nm, offered a mono-exponential decay with a lifetime of 4.86 ns, Fig. 11. Correspondingly, bi-exponential decay was observed upon adding varying concentrations of Ni-PTCA-SDS to an aqueous solution of TMPyP. This decay consists of an extremely fast component (80 ps, 15%), attributed to the electron transfer process within the TMPyP/Ni-PTCA-SDS complex, and a longer component (3.93 ns) comparable to the TMPyP reference (Fig. 11). Therefore, the rate of electron transfer (*k*_et_) and quantum yield (*Φ*_et_) were estimated to be 1.25 × 10^10^ s^−1^ and 0.98, respectively, based on lifetime values of TMPyP and TMPyP/Ni-PTCA-SDS composite. It is worth mentioning that the fluorescence lifetime of TMPyP is enhanced in the presence of SDS according to earlier studies, proving that the bi-exponential decay of TMPyP's fluorescence lifetime under the effect of Ni-PTCA-SDS is assigned to supramolecular electron transfer from electron-donating TMPyP to the electron-accepting Ni-PTCA-SDS composite. The thermodynamic driving force for photoinduced electron transfer was calculated to be −0.11 eV based on the oxidation potential of TMPyP and onset reduction potential of Ni-PTCA-SDS (−0.29 V *vs.* Ag/AgCl).^65^

**Fig. 11:**
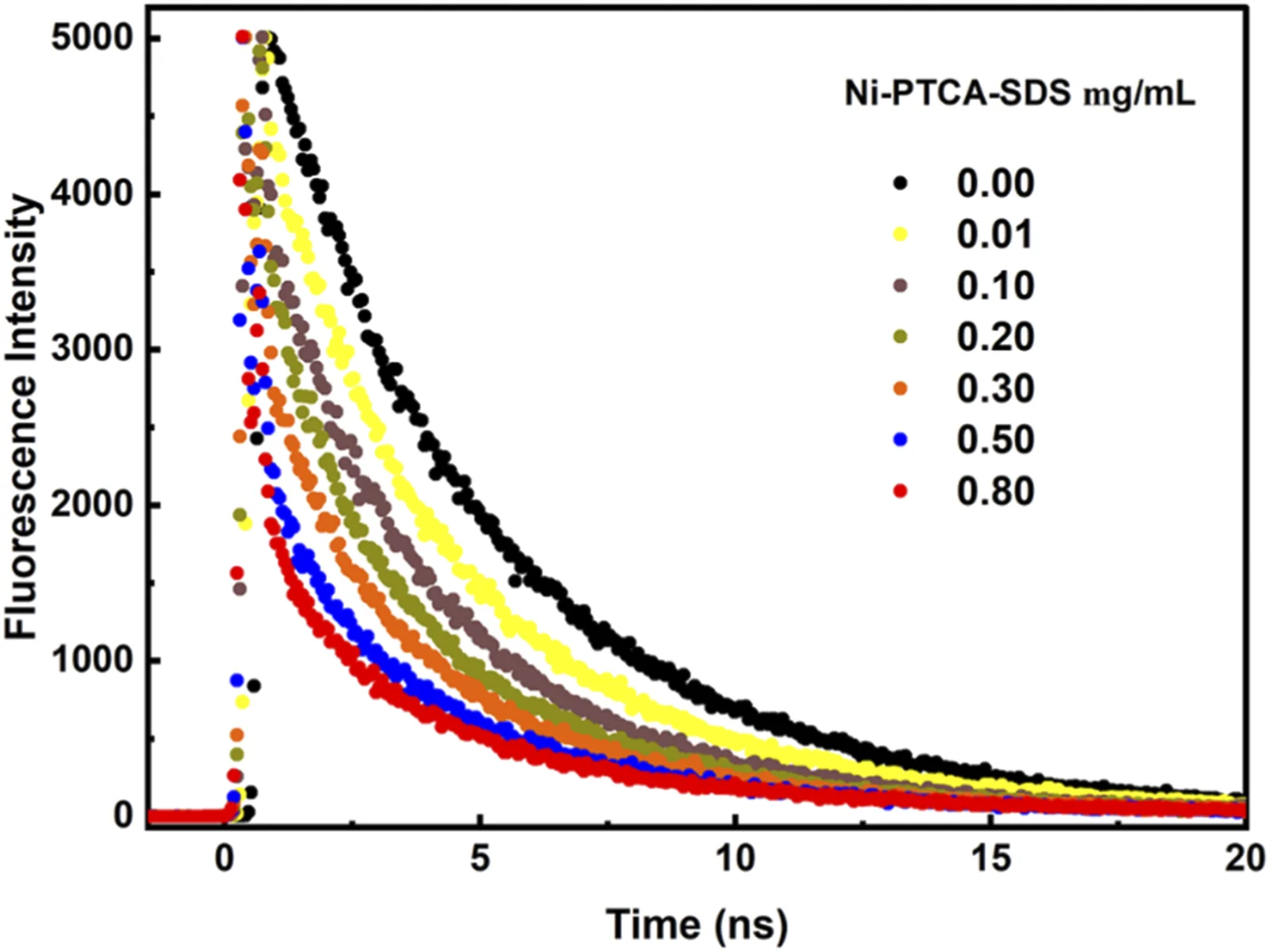
Fluorescence decay profiles of TMPyP (16.4 µM) with different Ni-PTCA-SDS additions (*λ*_ex_ = 421 nm, *λ*_em_ = 711 nm).

To conclude these photophysical studies (Section), when the hybrid system is irradiated under solar/broadband illumination, selective excitation of the perylene donor framework at 436 nm initiates FRET to the ground-state TMPyP acceptor. Subsequently, or upon direct excitation of the TMPyP Soret band (421 nm), ultra-fast intra-supramolecular photoinduced electron transfer occurs from the excited TMPyP to the Ni-PTCA-SDS acceptor framework (*k*_et_ = 3.26 × 10^11^ s^−1^). This demonstrates a synergistic energy-and-electron relay mechanism, where energy pooling feeds into efficient charge separation.

### 3.5. Electrochemical and photoelectrochemical evidence for interfacial charge transfer

To further investigate the electrochemical and photoinduced charge-transfer properties of the prepared materials, cyclic voltammetry (CV), electrochemical impedance spectroscopy (EIS), and chronoamperometric measurements were performed. These complementary measurements were used to assess the effect of Ni-PTCA-SDS formation and interfacial electron-transfer characteristics arise from subsequent electrostatic assembly of TMPyP on of the MOF system.

The cyclic voltammetry (CV) profiles shown in Fig. 12a reveal distinct cathodic responses for the investigated materials. The parent Ni-PTCA MOF exhibits a cathodic current density of approximately 4.9 mA cm^−2^, whereas Ni-PTCA-SDS displays an enhanced reduction current. This enhancement suggests improved electrochemical accessibility of the redox-active sites upon SDS modification of the MOF framework. More importantly, the TMPyP/Ni-PTCA-SDS composite exhibits the highest cathodic response among the materials investigated. This behavior can be associated with the intimate interfacial coupling between the π-conjugated TMPyP chromophore and the Ni-PTCA-SDS nanosheets, which provides an additional pathway for interfacial electron exchange. The enhanced cathodic response is therefore consistent with the proposed role of TMPyP as a photoactive electron donor and Ni-PTCA-SDS as an electron-accepting component within the supramolecular assembly.

**Fig. 12:**
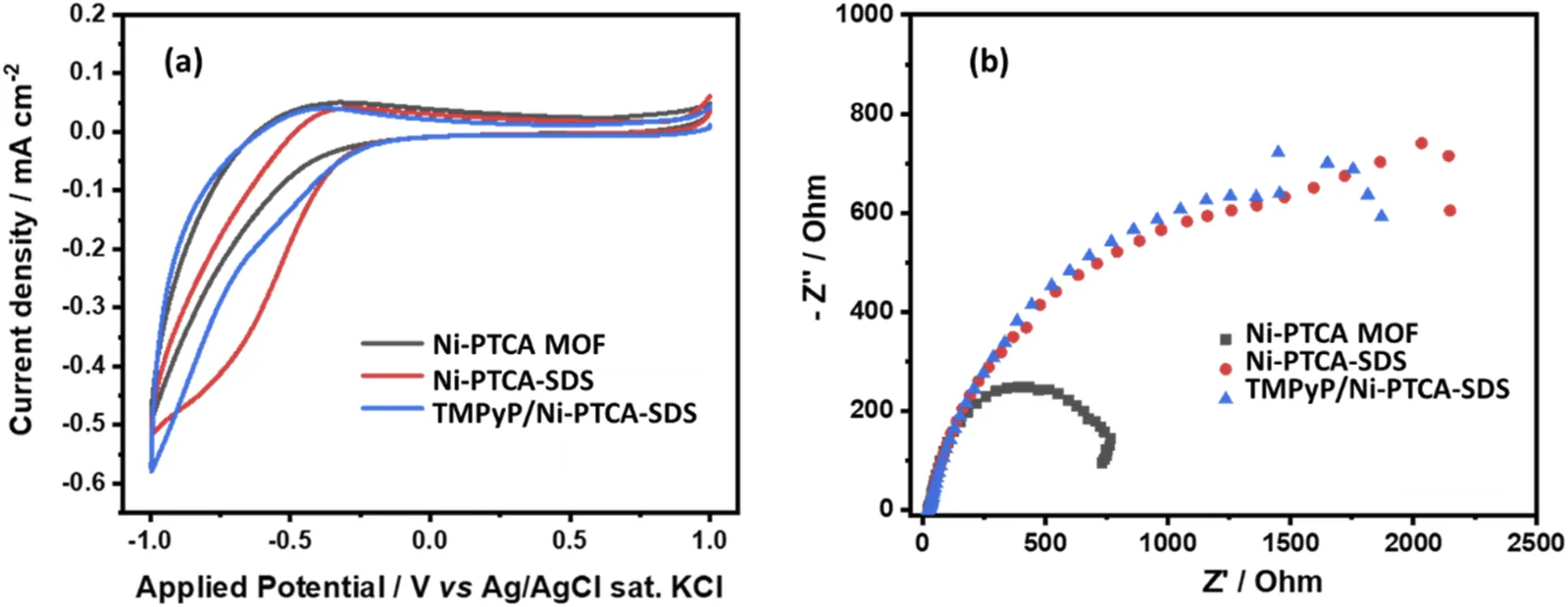
(a) Cyclic voltammetry of the examined materials with a potential window of 1 to −1 V *vs.* Ag/AgCl, Sat. KCl and a scan rate of 50 mV s^−1^, (b) electrochemical impedance spectroscopy (EIS) conducted for investigated materials in dark.

The electrochemical impedance spectroscopy (EIS) measurements provide further evidence for the interfacial charge-transfer dynamics within examined materials. Under dark conditions, both Ni-PTCA-SDS and TMPyP/Ni-PTCA-SDS exhibit larger Nyquist semicircles than the parent Ni-PTCA MOF, indicating increased charge-transfer resistance after modification of the parent framework (Fig. 12b). This behavior can reasonably be associated with the formation of additional interfacial components resulting from the framework modification of MOF and electrostatic assembly of TMPyP.

Upon light illumination, however, the charge-transfer resistance decreases substantially across all samples, demonstrating that photoexcitation promotes interfacial charge transport. Notably, as shown in Fig. 13a, the illuminated TMPyP/Ni-PTCA-SDS composite exhibits a lower charge-transfer resistance than Ni-PTCA-SDS. This impedance drop provides direct, independent electrochemical validation for the spectroscopic findings, confirming that TMPyP integration facilitates ultra-fast photoinduced interfacial charge transfer across the supramolecular system.

**Fig. 13:**
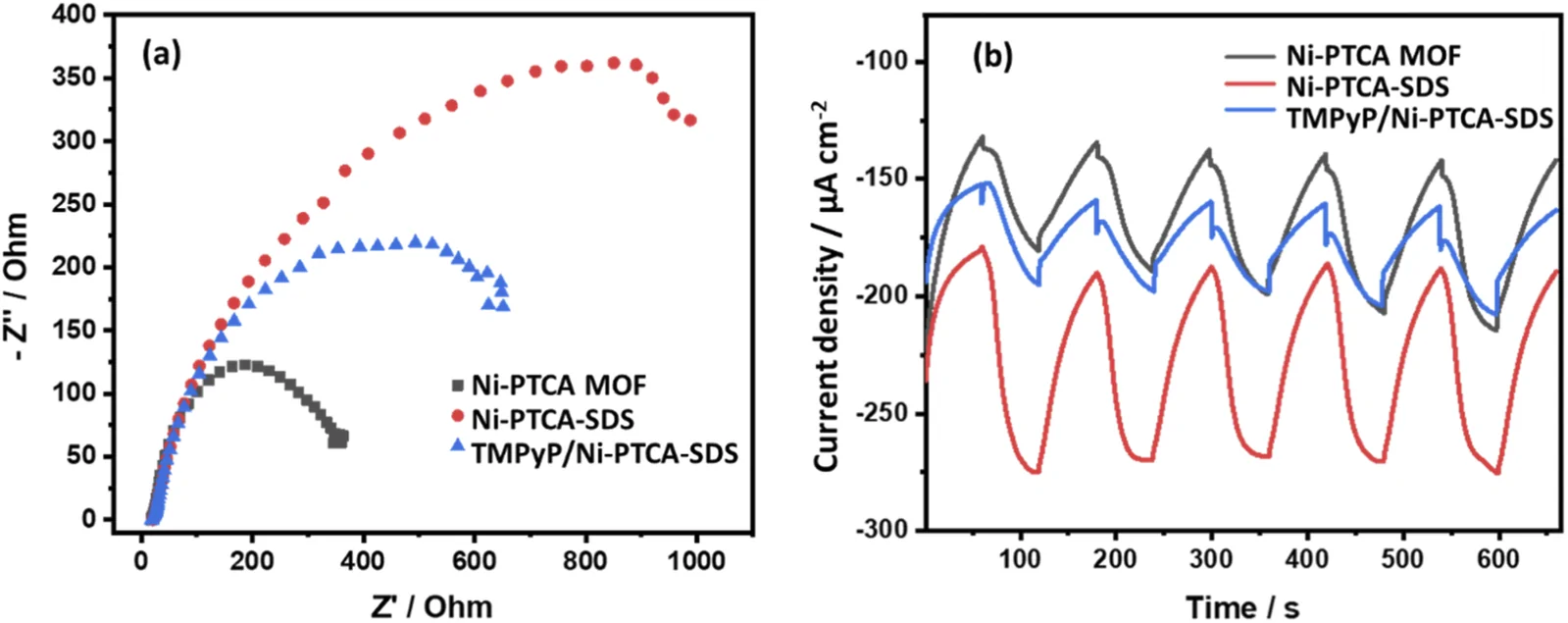
Photoelectrochemical measurements (a) electrochemical impedance spectroscopy (EIS) under light illumination, (b) chronoamperometry analysis (CA) of the examined materials.

The chronoamperometric measurements provide additional information concerning the photoresponse of the materials. At an applied potential of −0.8 V, Ni-PTCA-SDS exhibits the highest photocurrent amplitude, while TMPyP/Ni-PTCA-SDS also displays a pronounced and reproducible photoresponse (Fig. 13b). Although the higher photocurrent of Ni-PTCA-SDS indicates efficient generation and transport of photogenerated carriers under this polarization condition, photocurrent magnitude alone should not be considered a direct quantitative measure of charge-separation efficiency. The measured photocurrent is the net result of photon absorption, carrier generation, interfacial electron transfer, carrier trapping, recombination, and the direction of charge extraction imposed by the applied potential.

This point becomes particularly evident from the additional chronoamperometric experiment performed at +1.0 V (Fig. 14). At this potential, both Ni-PTCA-SDS and TMPyP/Ni-PTCA-SDS exhibit substantially stronger photoresponses than the parent Ni-PTCA MOF. The response of TMPyP/Ni-PTCA-SDS is particularly pronounced during the initial illumination period, while Ni-PTCA-SDS also exhibits a strong and reproducible photoresponse. In contrast, Ni-PTCA MOF produces a considerably weaker photocurrent response throughout the measurement. Therefore, the measurements at +1.0 V provide clear evidence that conversion of the parent MOF into Ni-PTCA-SDS markedly enhances its photoelectrochemical activity and that the incorporation of TMPyP preserves a strong photoinduced charge-transport response. In addition, at an applied potential of +1.0 V, the transient photocurrent measurements exhibited a decrease in the background current during the initial stage of testing. However, the photocurrent response remained reproducible during successive 60 s dark/60 s illumination cycles, with no noticeable deterioration in the light-induced current response. This behavior suggests that the observed overall current decrease is primarily associated with changes in the background current rather than a pronounced loss of photoactivity.

**Fig. 14:**
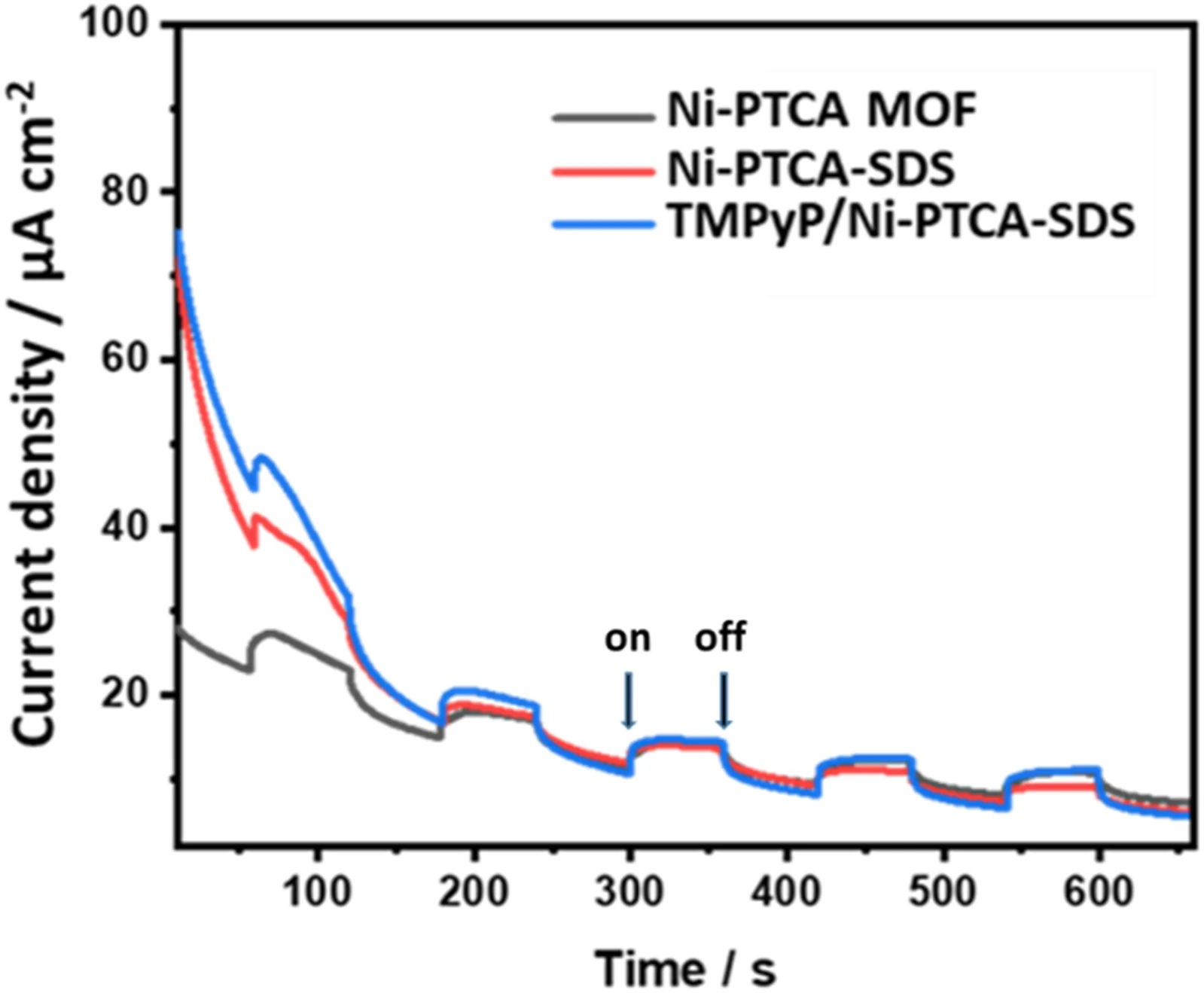
Chronoamperometric photocurrent responses of Ni-PTCA MOF, Ni-PTCA-SDS, and TMPyP/Ni-PTCA-SDS recorded at an applied potential of +1.0 V under repeated light on/off cycles.

The comparison between Fig. 13b and 14 is particularly informative because it demonstrates that the relative photocurrent responses depend on the applied potential. Whereas Ni-PTCA-SDS gives the largest response at −0.8 V, TMPyP/Ni-PTCA-SDS exhibits a strong photoresponse at +1.0 V and remains highly photoresponsive under the different polarization conditions. This potential-dependent behavior indicates that the applied bias influences the preferential extraction and transport of photogenerated carriers at the electrode/electrolyte interface. Consequently, the photoelectrochemical behavior of these materials cannot be adequately described simply by ranking the samples according to a single photocurrent value.

The strong response of TMPyP/Ni-PTCA-SDS at +1.0 V is also consistent with the proposed light-harvesting function of TMPyP. Incorporation of the porphyrin chromophore extends the photoactive supramolecular system and provides an additional pathway for generation and transfer of excited-state charge carriers. Importantly, the spectroscopic investigations independently demonstrated highly efficient photoinduced electron transfer from TMPyP to Ni-PTCA-SDS, with an electron-transfer efficiency exceeding 95% and a rate constant of 3.26 × 10^11^ s^−1^. In parallel, Förster resonance energy transfer from excited Ni-PTCA-SDS to TMPyP was observed with an efficiency of approximately 69%. These simultaneous electron- and energy-transfer processes provide a plausible explanation for the strong and potential-dependent photoelectrochemical response of the composite.

The transient photocurrent profiles further exhibit rapid changes following light switching, followed by slower relaxation toward the baseline. The initial rapid decay after removal of illumination can be attributed to the depletion/recombination of relatively mobile photogenerated carriers, whereas the slower component is consistent with the relaxation of longer-lived trapped or interfacial stored charge carriers. Similar fast and slow decay components were observed in the photoresponse behavior of the MOF-based systems. The reproducible switching behavior observed in Fig. 13b and 14 additionally confirms the reversible photoresponse of the electrodes.

Overall, the CV, EIS, and chronoamperometric measurements provide complementary evidence for the modification of charge-transfer mobility within MOF in Ni-PTCA-SDS and photoinduced electron transfer occurs from porphyrin (TMPyP) to the Ni-PTCA-SDS nanosheets. The CV results demonstrate enhanced electrochemical accessibility and interfacial electron exchange, while EIS shows that illumination significantly lowers the charge-transfer resistance, particularly for TMPyP/Ni-PTCA-SDS. The chronoamperometric measurements further demonstrate that Ni-PTCA-SDS and TMPyP/Ni-PTCA-SDS possess substantially stronger photoresponses than the parent Ni-PTCA MOF, particularly at +1.0 V (Fig. 14). The difference in photocurrent magnitude at −0.8 and +1.0 V further emphasizes the potential-dependent nature of carrier extraction rather than indicating a simple difference in intrinsic charge-separation efficiency.

## 4. Conclusion

In summary, a novel porphyrin/perylene-based nickel-MOF hybrid has been successfully fabricated to serve as an efficient platform for interfacial energy and electron transfer relays. Comprehensive physicochemical characterization—including XRD, FTIR, DLS, zeta potential, SEM, and HR-TEM—confirmed the successful construction and robust electrostatic self-assembly of uniform TMPyP/Ni-PTCA-SDS composite nanosheets in an aqueous medium. Steady-state and time-resolved fluorescence analyses demonstrated rapid and efficient photoinduced charge separation from the electron-donating TMPyP to the electron-acceptor Ni-PTCA-SDS framework, reinforced by a synergistic energy transfer mechanism. This process yielded an exceptional electron transfer rate of 1.25 × 10^10^ s^−1^ with high efficiency of 0.98. Furthermore, photoelectrochemical evaluations, including cyclic voltammetry, electrochemical impedance spectroscopy, and transient photocurrent measurements, provided strong evidence of significantly enhanced interfacial charge transport and minimized charge-transfer resistance under illumination. Ultimately, this study highlights the profound impact of well-defined MOF-based hybrid architecture on charge-separation material/photoelectrode component that lays a robust foundation for future solar-to-chemical applications.

## Conflicts of interest

The authors declare that they have no known competing financial interests or personal relationships that could have appeared to influence the work reported in this paper.

## Supplementary Material

RA-OLF-D6RA09222E-s001

## Data Availability

Data will be made available on request. Supplementary information (SI) is available. See DOI: https://doi.org/10.1039/d6ra09222e.
