# Supplementary material for: Porphyrin-decorated perylene–nickel-MOF nanocomposite: photoinduced electron and energy transfer for photoelectrochemical applications
Source: RSC Adv. 2026 Nov 9. Online ahead of print. doi: 10.1039/d6ra09222e (PMC13747202; doi:10.1039/d6ra09222e)
Supplement: Supplementary file 1 [file RA-OLF-D6RA09222E-s001.pdf]

Supplementary Information (SI) for RSC Advances.
This journal is © The Royal Society of Chemistry

Supplementary Materials

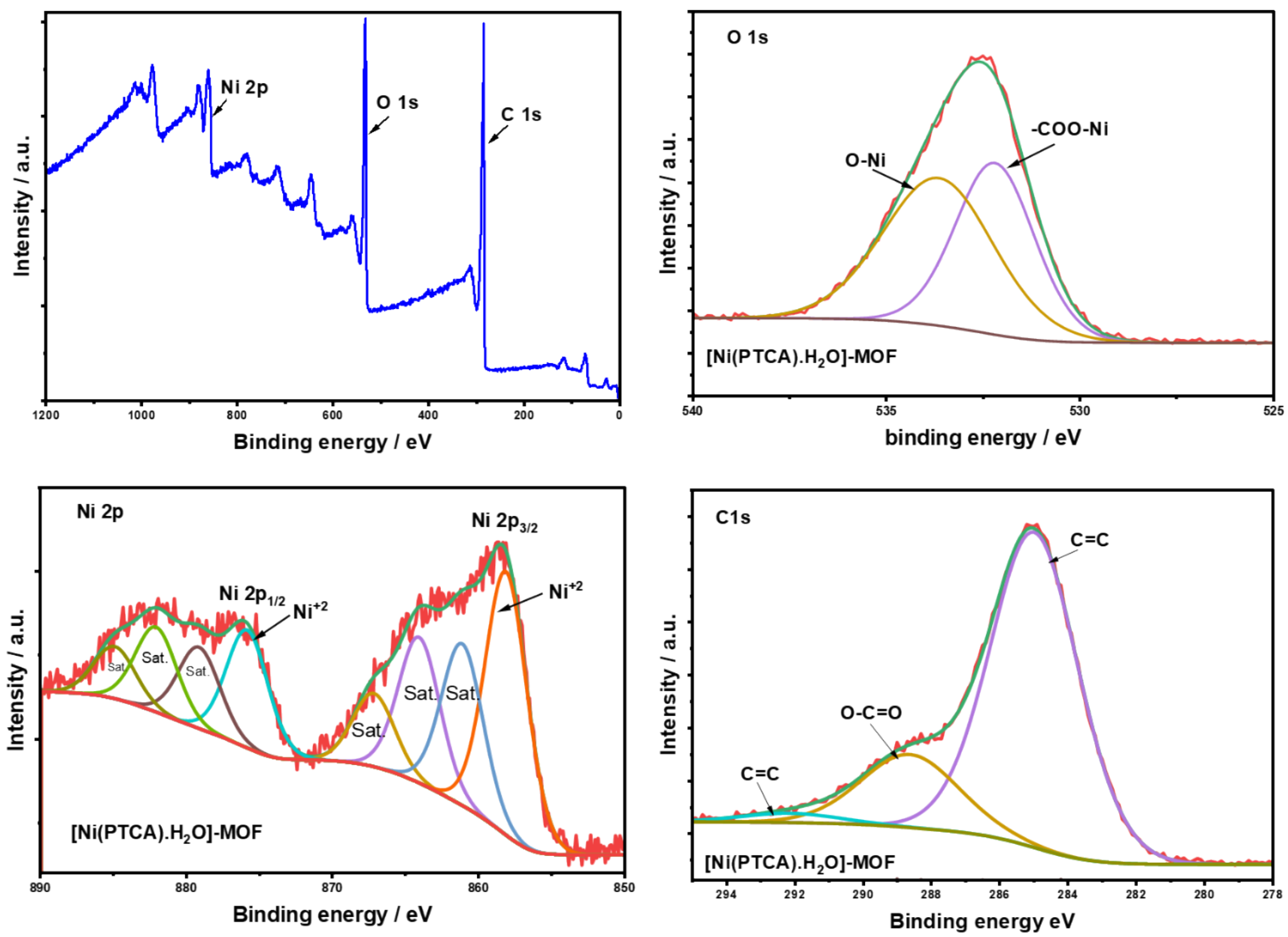

Fig. S1. XPS element peaks of Ni 2p, O 1s, and C1s and survey of Ni-PTCA MOF.

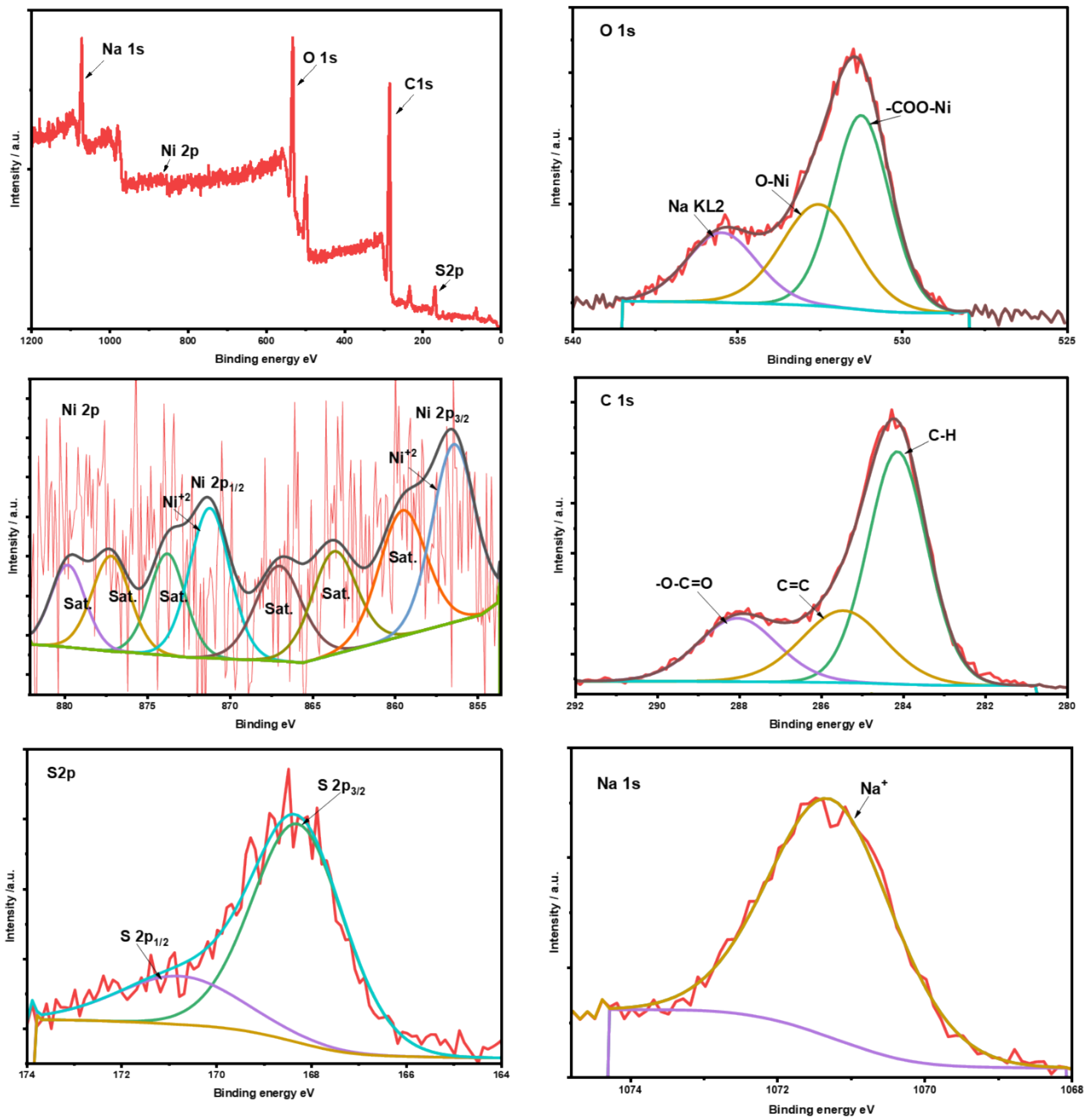

Fig. S2. XPS element peaks of Ni 2p, O 1s, C 1s, S 2p, and Na 1s and survey of Ni-PTCA-SDS.

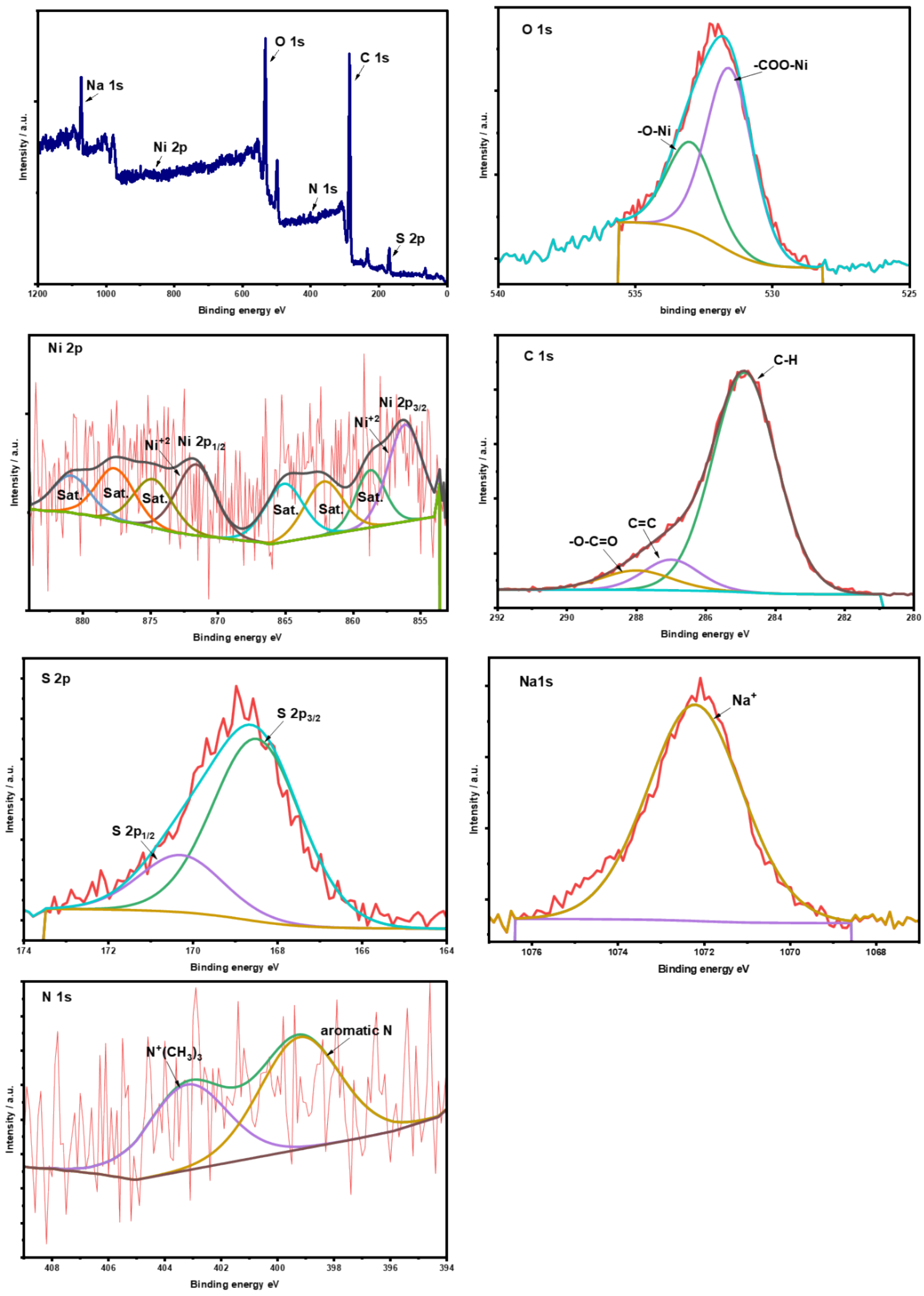

Fig. S3. XPS element peaks of Ni 2p, O 1s, C 1s, S 2p, Na 1s, and N 1s and survey of TMPyP/Ni-PTCA-SDS.

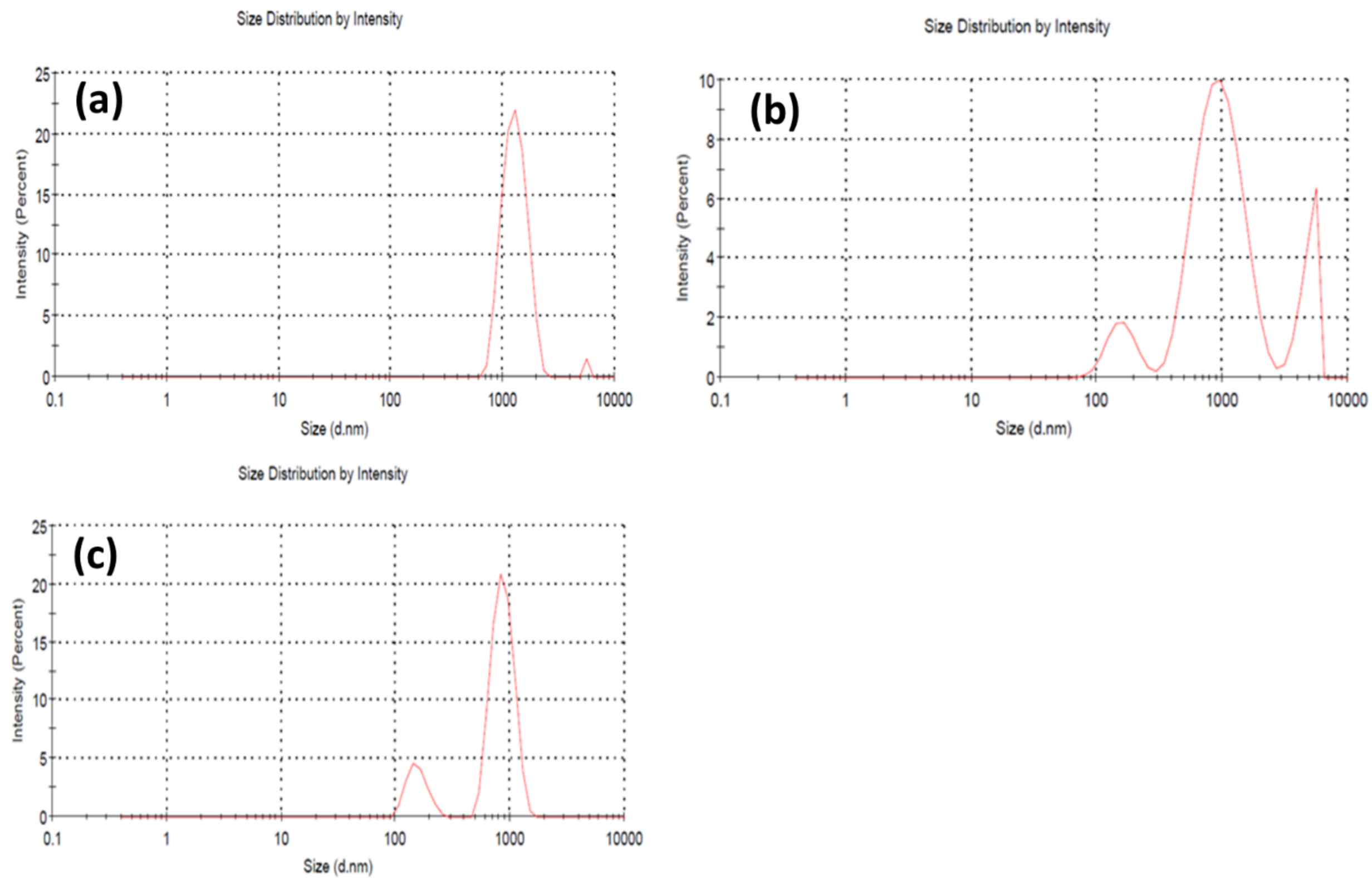

Fig. S4. Hydrodynamic size distribution of (a) Ni-PTCA MOF (b) Ni-PTCA-SDS (c) TMPyP/Ni-PTCA-SDS obtained from DLS measurements.

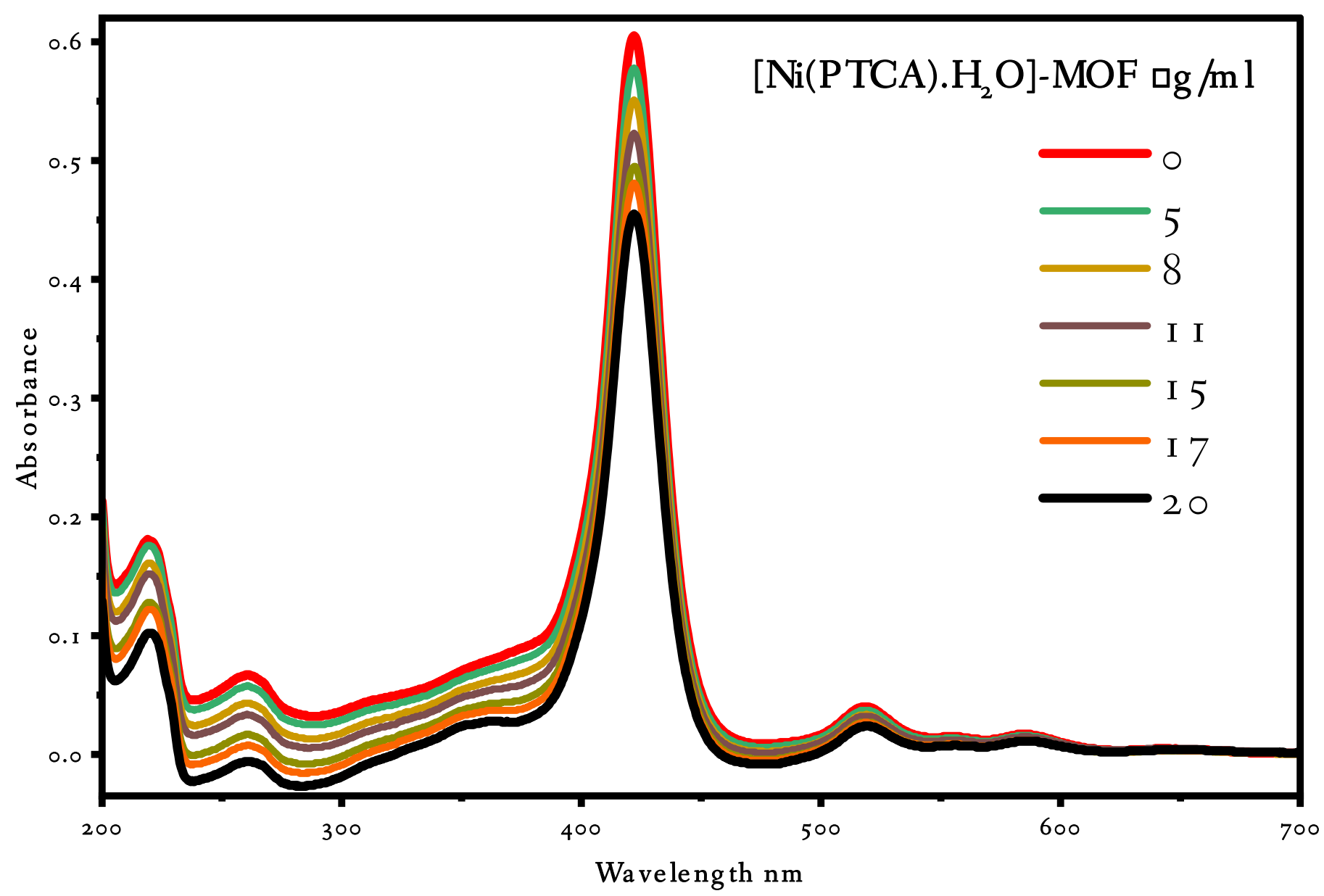

Fig. S5. Absorption spectra of TMPyP (16.4 x 10^{-4} mM) during the addition of various Ni-PTCA MOF concentrations.

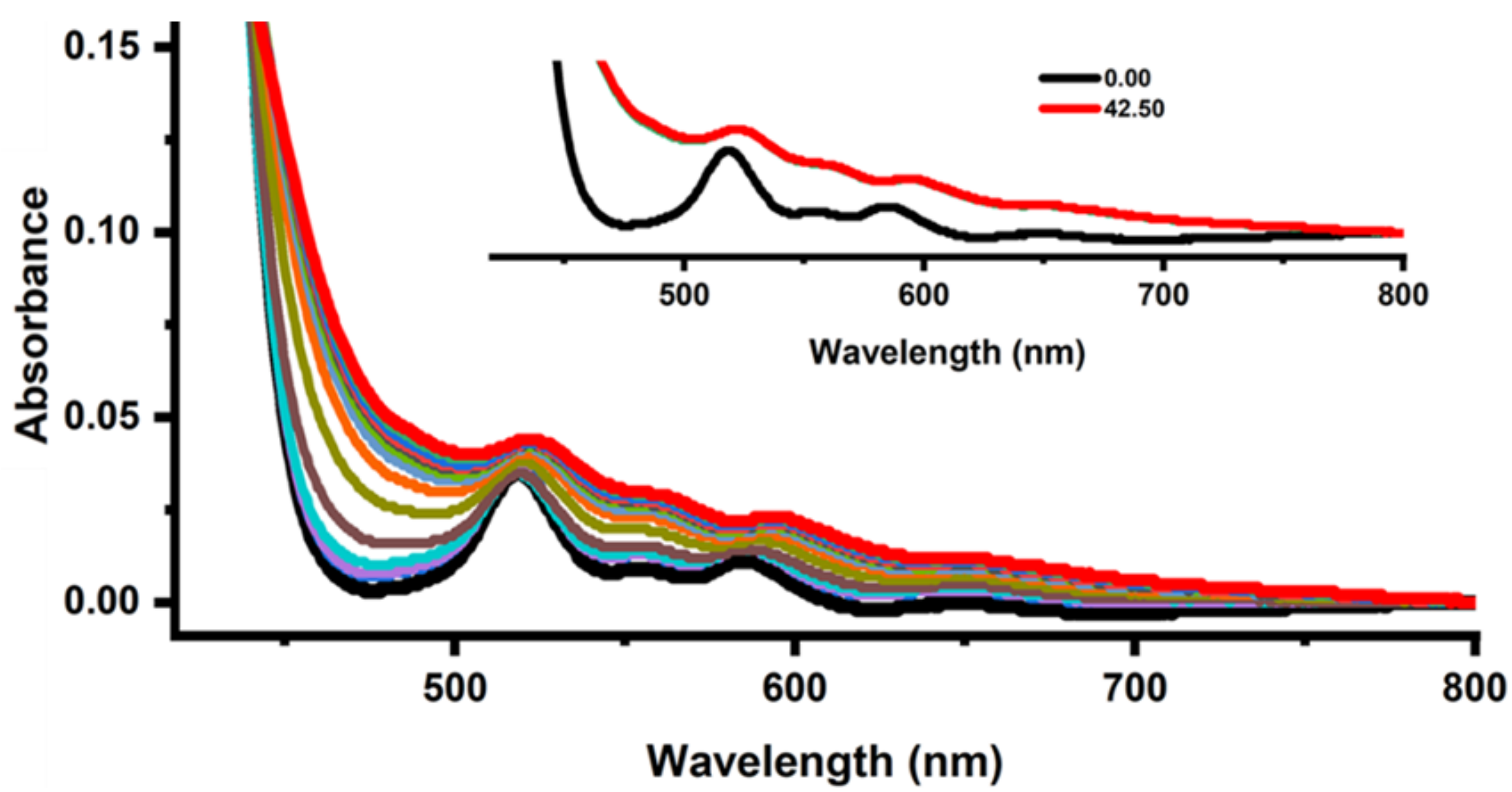

Fig. S6. Absorption spectral change of absorption of four weak Q vibronic bands at 517, 554, 585 and 648 nm corresponding to TMPyP (16.4 x 10^{-4} mM) during the addition of various Ni-PTCA-SDS concentrations.

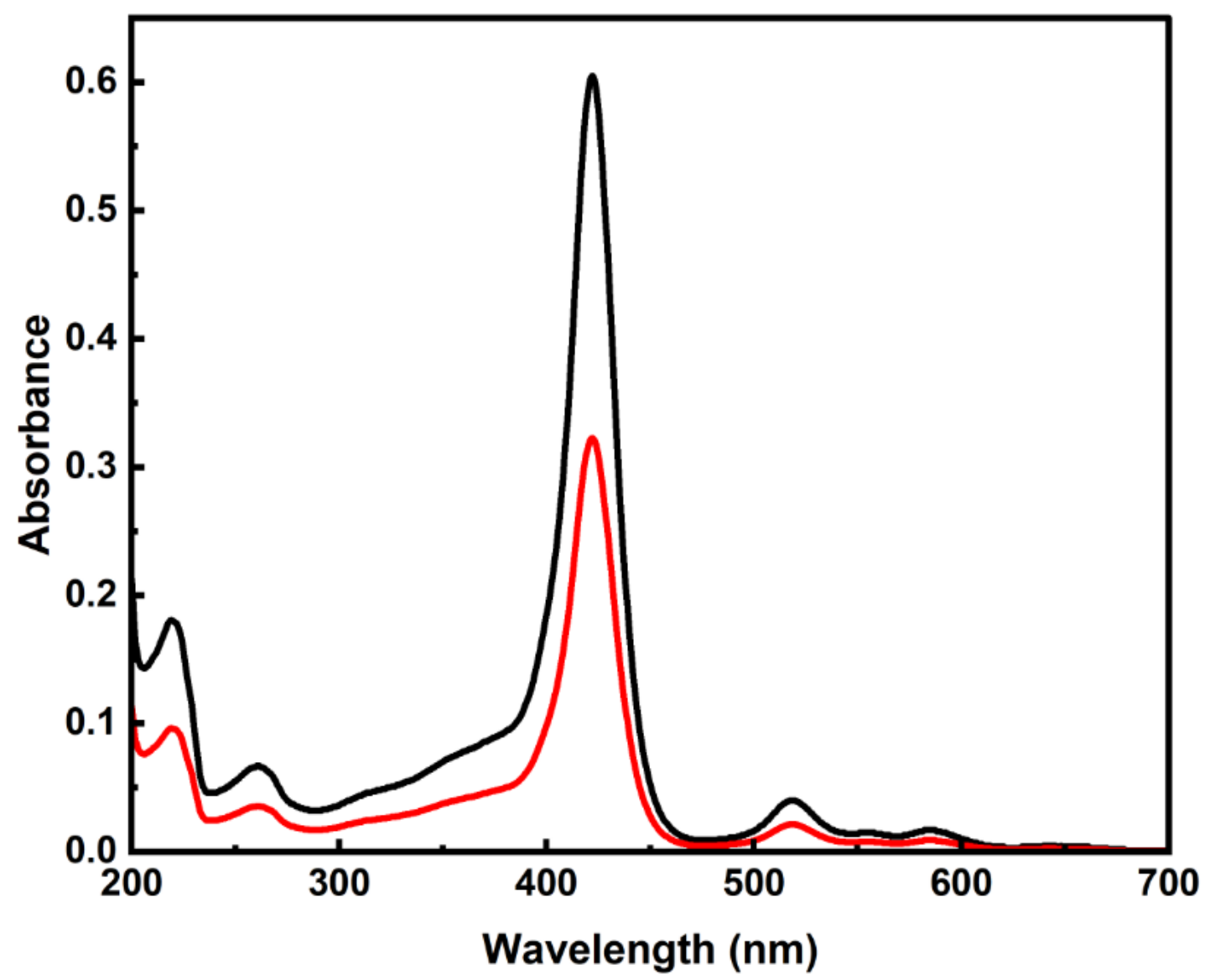

Fig. S7. Absorption spectra of a known initial concentration TMPyP solution (black line) and its absorption in the supernatant (red line) after 24 hr stirring and isolation of Ni-PTCA-SDS.

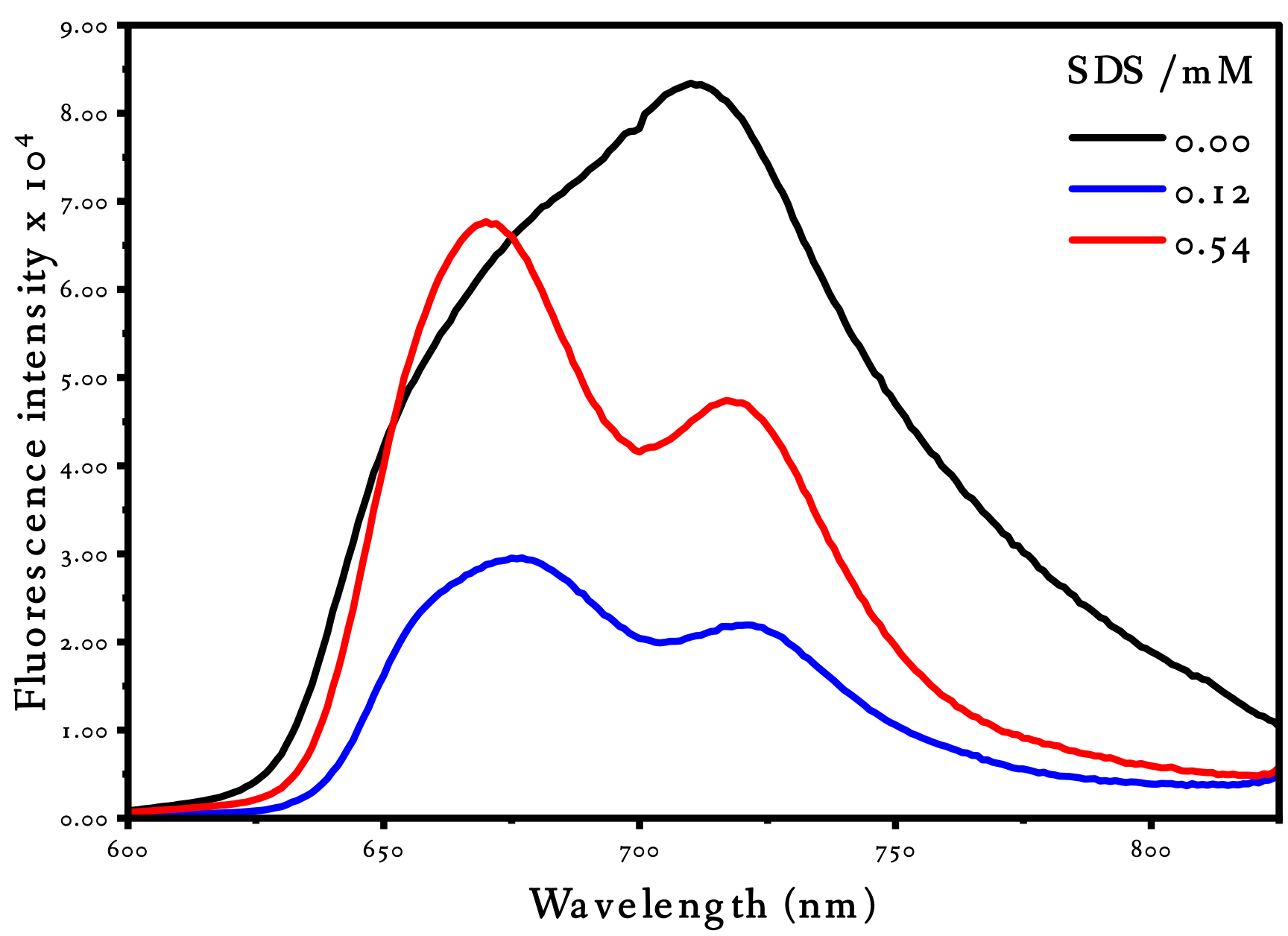

Fig. S8. Spectral effect of SDS addition on TMPyP (16.4×10^{-4} mM) emission spectra.

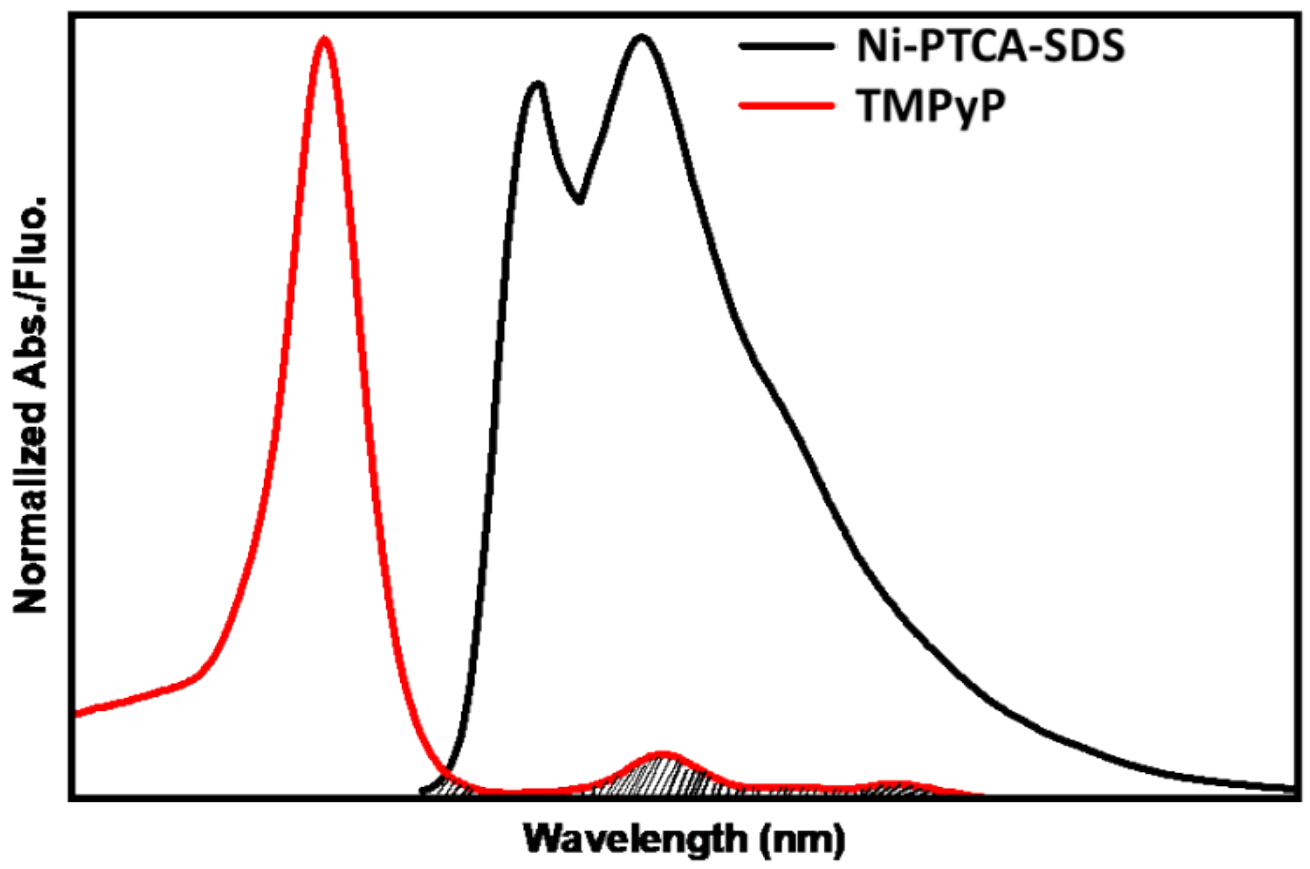

Fig. S9. Spectral overlap between TMPyP absorption and Ni-PTCA-SDS emission.

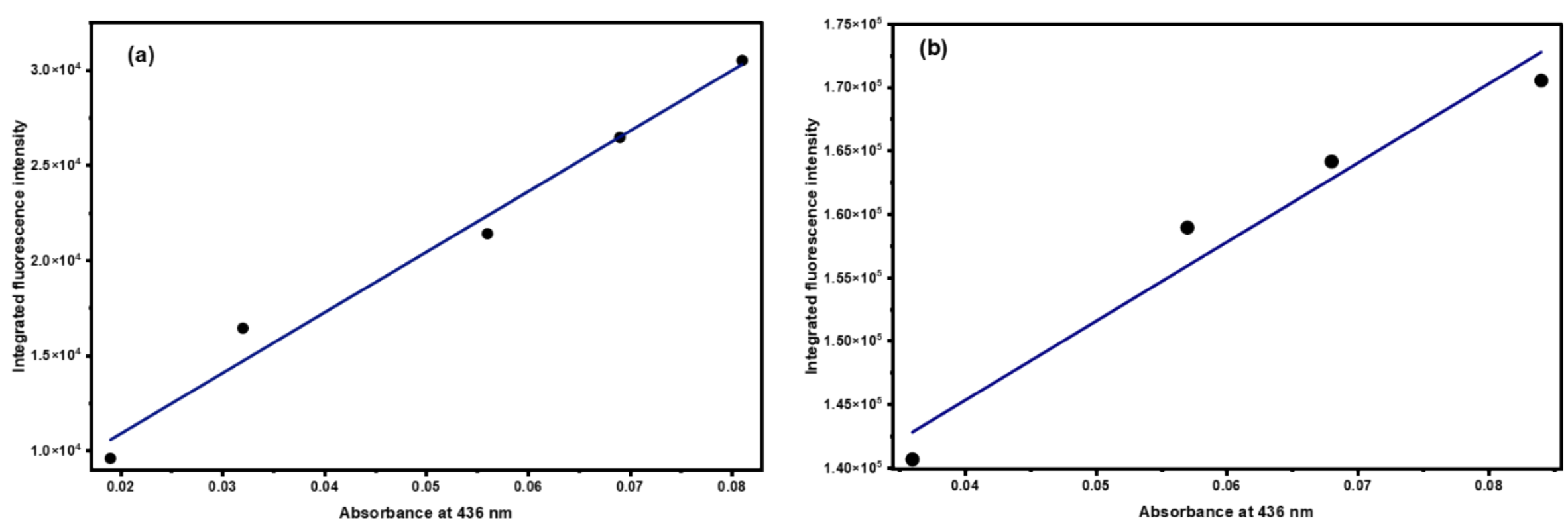

Fig. S10. (a) Linear relationship between integrated fluorescence emission intensity of Ni-PTCA-SDS and its absorbance at 436 nm. (b) linear relationship between the integrated fluorescence emission intensity of reference (TAIPDI) and its absorbance at 436 nm.
